# Thermal Cross Linking of Novel Azide Modified Polymers of Intrinsic Microporosity—Effect of Distribution and the Gas Separation Performance

**DOI:** 10.3390/polym11081241

**Published:** 2019-07-26

**Authors:** Silvio Neumann, Gisela Bengtson, David Meis, Volkan Filiz

**Affiliations:** Helmholtz-Zentrum Geesthacht, Institute of Polymer Research, Max-Planck-Straße 1, 21502 Geesthacht, Germany

**Keywords:** polymers of intrinsic microporosity, crosslinking, azide, gas separation, membranes

## Abstract

The synthesis of polymers of intrinsic microporosity (PIM) modified with azide groups, the cross linkage by nitrene reaction and their performance as gas separation membranes are reported. The azide modification of the spirobisindane units in the polymer backbone was done by post functionalization of methylated spirobisindane containing polymers. These polymers differ in distribution and concentration of the azide group containing spirobisindane units by applying perfectly alternating and randomly distributed copolymers along the polymer chains. To investigate the influence of concentration of the azide groups, additionally the homopolymer of methylated spirobisindane was synthesized and subjected to identical treatments and characterizations as both copolymers. Cross linkage by nitrene reaction was examined by different temperature treatments at 150, 200, 250 and 300 °C. Characterization of the new polymers was performed by NMR, SEC and FT-IR. Furthermore, the crosslinking process was investigated by means of solid state NMR, TGA-FTIR, DSC and isoconversional kinetic analysis performed with TGA. Gas permeability of CO_2_, N_2_, CH_4_, H_2_ and O_2_ was determined by time lag experiments and ideal selectivities for several gas pairs were calculated. The two azide groups per repeating unit degrade during thermal treatments by release of nitrogen and form mechanically stable PIM networks, leading to an increase in gas permeability while selectivity remained nearly constant. Measured diffusivity and solubility coefficients revealed differences in the formation of free volume elements depending on distribution and concentration of the azide groups. Aging studies over about five months were performed and physical aging rates (β_P_) were evaluated with regard to the concentration and distribution of curable azide functionalities. Subsequently, the enhanced sieving effect during aging resulted in membrane materials that surpassed the Robeson upper bound in selected gas pairs.

## 1. Introduction

Microporous materials have been of great interest for many applications in fields such as clean energy, catalysis and storage media due to their extraordinarily high porosity and surface area. Following the recent definition from the International Union of Pure and Applied Chemistry (IUPAC), microporous materials involve extremely small pores with average sizes of less than 2 nm [1]. Moreover, the easy processability of microporous polymers has demonstrated a great advantage with respect to the scale-up for commercial applications [2,3,4]. Over the last 40 years, substantial studies on polymeric membrane materials have been executed with the goal to improve their gas separation performance. In this context, gas separation performance is described by gas permeability and selectivity. Consequently, both permeability and selectivity, have been improved compared to first generation materials [5,6]. However, the performance of current materials still shows a limitation, when it comes to the improvement of both permeability and selectivity at the same time. This limitation is called the “upper-bound” [7]. Assembling a large number of data points from previous references, L. M. Robeson introduced an “upper-bound” regarding the trade-off between gas permeability and selectivity for valuable gas pairs. This trade-off relationship of membrane materials describes well that highly permeable materials usually show low separation properties and *vice versa*. Microporous polymers, such as substituted polyacetylenes and amorphous fluoropolymers have demonstrated higher permeabilities than other low free volume polymers. However, due to the trade-off relationship, they exhibit a low selectivity, respectively [5]. 

In the last decade, new classes of microporous polymers, such as thermally rearranged (TR) polymers and polymers of intrinsic microporosity (PIM), have been reported showing good selectivities, as well as extraordinary gas permeabilities. The performance of these polymers as membrane materials surpassed the upper-bound and have provided the potential for a more energy efficient membrane-based gas separation process for several industrial applications [8,9]. Unfortunately, TR polymers as well as PIMs possess serious disadvantages. In the case of TR polymers, a narrow distribution of free volume elements or cavity sizes is mainly derived from a post-thermal conversion of functionalized polyimides at about 400 °C. This high temperature treatment, although it meanwhile could be reduced to approximately 300 °C by chemical modification, impedes the use of these materials in the field of membrane applications. [10]. In PIMs, due to their contorted and rigid molecular structure as well as the accompanied hindered packing of polymer chains, free volume elements are formed. However, the gas permeability decreases with time as a result of physical aging, which is primarily due to the collapse of larger pores by compaction [11]. This last assessment by Staiger was supported further by a work by Bernardo and co-workers, who concluded that the decrease in permeability is entirely controlled by diffusivity [12]. 

Since physical aging originates from chain flexibility and the non-equilibrium state of glassy polymers like PIM-1, several approaches try to mitigate this undesirable phenomenon by increasing polymer chain rigidity or by inter-chain cross linking [13]. Regarding the increase in rigidity of the polymer chain, attempts were executed by introduction of new monomeric units in the polymer backbone or by post-modification of PIM-1 and PIM-like structures [14,15,16,17,18,19,20,21,22,23,24]. Crosslinking of the PIM-1 backbone seems not that prominent. Some attempts were made with the help of UV radiation, which mostly led to a rearranged polymer backbone instead of forming interconnections between the polymer chains [25,26,27]. Chemical cross linking was performed by addition of pyrenes or azidic compounds to link PIM chains by thermal treatments as discussed by Song and Li [28,29,30]. In case of an unmodified PIM-1 Song and Li had shown that especially the nitrile group of the polymer is reactive at high temperatures with respect to the formation of linkages between the polymer chains [26,31]. Du also presented a thermal cross linking, the nitrile group of PIM-1 was converted to carboxylic acid groups prior to temperature treatments [32]. These approaches have in common that the nitrile group is sacrificed, non-regarding the observation, especially in the beginning of the investigation of PIM-1, that the nitrile groups were described as an important functionality that enhance strength of intermolecular forces and the free volume because of their lateral position, [33,34]. 

This work presents an approach to crosslink polymers of intrinsic microporosity without modifying the nitrile group, in order to maintain the characteristics of its functionality. Here, the methylated spirobisindane unit was modified by simple and fast chemical reactions to yield an azide group in benzyl position. Azide groups are well known to decompose at rather low temperatures (approx. 200 °C). As a result of the cleavage of the azide group, nitrogen is evolved to generate a highly reactive nitrene radical. This creates various interconnections between the PIM chains depending on different temperature treatments. The resulting structures are verified and gas separation performance as well as the physical aging behavior are studied.

## 2. Materials and Methods

### 2.1. Materials

The monomer 5,5′,6,6′-tetrahydroxy-3,3,3′,3′-tetramethyl-1,1′-spirobisindane (TTSBI, 97%) was obtained from ABCR (Karlsruhe, Germany) and 2,3,5,6-tetrafluoroterephtalonitrile (TFTDN) was kindly donated by Lanxess (Köln, Germany). TFTDN was sublimated twice at 80 °C and < 1 mbar prior to use. Potassium carbonate (K_2_CO_3_, >99,5%) was dried overnight at 120 °C under vacuum and then milled in a ball mill for 15 min.

For the synthesis of dimethyl-TTSBI (DMTTSBI) 3-methylcatechol, hydro bromic acid (HBr, 47% in H_2_O) and acetic acid (HAc) were obtained from Sigma Aldrich. As well as benzoylperoxide (BPO, 75%), diethylbenzene (DEB), chlorobenzene (anhydrous) and sodium azide (99%) for further reactions. They were used as received. *N*-bromosuccinimide (*N*BS, ≥99%), *N,N*-dimethylacetamide (DMAc, ≥99.8%), *N,N*-dimethylformamide (DMF, ≥ 99.8%), chloroform (CHCl_3_, 99.9%), methanol (CH_3_OH, ≥99%) and acetone (≥99%) were obtained from Merck and used as received.

### 2.2. Synthesis of Monomer DMTTSBI and Trimer

#### 2.2.1. Synthesis of DMTTSBI

The monomer 7,7′-Dimethyl-5,5′6,6′-tetrahydroxy-3,3,3′,3′-tetramethyl-1,1′-spirobisindane (DMTTSBI) was prepared following a reaction route proposed by Fritsch et al. [35]. 3-methylcatechol (20.0 g, 160.00 mmol) was suspended in HAc (40 mL) followed by the addition of aqueous HBr (45 mL, 840.00 mmol) which resulted in a clear solution. Gradual addition of acetone (26 mL, 350.00 mmol) over 30 min formed a dark brown solution which was further heated at 120 °C for 12 h for complete reaction. After cooling, DMTTSBI was isolated by precipitation in 2 liters of water and left for stirring overnight. Post filtration, the monomer was washed several times with water until the pH of the filtrate reached 5. The monomer was further cleaned by washing several times with diethyl ether, resulting in a white powder. Drying in vacuum at 40 °C yielded 12.2 g (46%) of DMTTSBI, m.p. 230 °C.

#### 2.2.2. Synthesis of Trimer

For the synthesis of an alternating polymer, it was necessary to prepare a trimer consisting of one TTSBI molecule and two TFTDN molecules (Figure S1). Therefore, TFTDN (3.60 g, 18.00 mmol) was dissolved in DMF (30 mL). Afterwards K_2_CO_3_ (1.69 g, 12.24 mmol) was added and the mixture was heated to 55 °C. TTSBI (2.04 g, 6.00 mmol) was dissolved in DMF (10 mL) separately and after complete dissolution, added dropwise to the TFTDN solution over 30 minutes. After 24 hours, the reaction mixture was poured in 600 mL water, the yellow precipitate was filtered off and dried over night at 60 °C in vacuum. To remove residual K_2_CO_3_ the light yellow solid was dissolved in THF and precipitated again in water. To remove residual TFTDN the trimer was stirred in acetone for several hours and filtrated. The material was dried at 60 °C overnight. As a consequence of the stoichiometric importance of residual traces of TFTDN, the trimer was additionally treated at 80 °C in low pressure. This procedure yielded 2.47 g of light yellow powder (3.74 mmol, 62%), m.p. 370 °C. 

*^1^H-NMR (500 MHz, Methylene Chloride-d_2_, Figure S3 above) δ 6.94 (s, 2H), 6.54 (s, 2H), 2.44 (d, J = 13.2 Hz, 2H), 2.23 (d, J = 13.2 Hz, 2H), 1.43 (s, 6H), 1.37 (s, 6H). ^13^C-NMR (126 MHz, CD_2_Cl_2_, Figure S4 above) δ 145.83, 145.69, 144.44, 112.44, 110.65, 96.51, 96.45, 96.29, 64.83, 60.69, 53.55, 39.86, 33.09, 31.57, 31.03, 30.59, 29.51, 28.98, 24.84, 23.34, 19.15. ^19^F-NMR (282 MHz, DMF-d_7_, Figure S3 below) δ -140.63. SEC (CHCl_3_): M_w_ = 722 g/mol D = 1005. FT-IR (ATR, Figure S4 below): 2960, 2920, 2864, 2242, 1470 (s), 1407, 1311, 1265, 1213, 1122, 1110, 1006 (s), 945, 878, 753, 650 cm^−1^.*

### 2.3. Synthesis of Homopolymer and Copolymers

#### 2.3.1. Synthesis of Homopolymer and Randomly Distributed Copolymer

**Figure 1:**
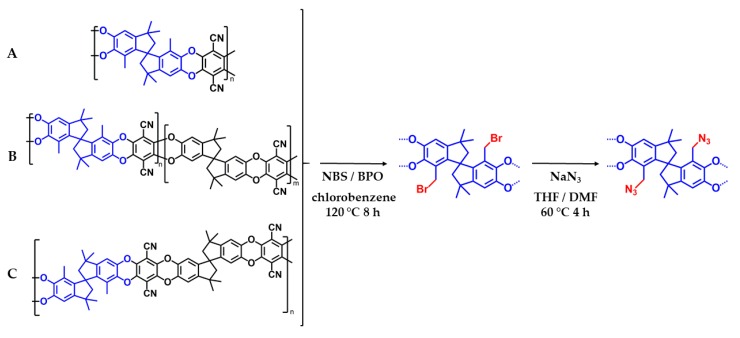
Modification route for the preparation of azide modified polymers of intrinsic microporosity (A: homopolymer—AZ-PIM-100, B: randomly distributed DMTTSBI unit—AZ-PIM-R50 and C: alternating DMTTSBI unit—AZ-PIM-A50).

Synthesis of homopolymer (PIM-DMTTSBI-100) and randomly distributed DMTTSBI copolymer (PIM-DMTTSBI_R50) (Structures see Figure 1). Polymers were prepared similar to the fast synthetic route developed for the synthesis of PIM-1 (Figure S2) [36]. For preparation of the homopolymer equimolar amounts of DMTTSBI and TFTDN and for the copolymer adapted molar quantities of DMTTSBI, TTSBI and TFTDN (1:1:2), were dissolved in DMAc, resulting in an orange-brownish solution. The addition of potassium carbonate started the polymerization reaction after putting the flask in a hot oil bath at 150 °C and caused a color change to yellow. After 45 min the reaction was stopped by precipitation in methanol. The polymer was filtrated and dried in vacuum at 100 °C for about 24 h. The yellow solids were cleaned by reprecipitation from THF in water, after filtration and drying, again from chloroform solution in methanol and finally by stirring in acetone for several hours. Subsequent drying at 100 °C for 24 h in vacuum resulted in yellow polymers (yield 92 resp. 96%). The polymers were characterized by ^1^H-NMR, FT-IR and SEC (Table S1).

*PIM-DMTTSBI-100: ^1^H-NMR (500 MHz, Chloroform-d, Figure S11) δ 6.71 (s, 2H), 2.09–2.34 (m, 4H), 1.74 (s, 6H), 1.35 (s, 6H), 1.31 (s, 6H). FTIR (ATR, Figure S12): 2956, 2867, 2239, 1711, 1612, 1573, 1444 (s), 1324, 1293, 1265 (s), 1214, 1197, 1166, 1052 (s), 998, 857, 752 cm^−1^.*

*PIM-DMTTSBI-R50: ^1^H-NMR (500 MHz, Chloroform-d, Figure S8) δ 6.82 (s, 2H), 6.70 (s, 2H), 6.42 (s, 2H), 2.70–1.98 (m, 8H), 1.92–1.62 (m, 6H), 1.34 (d, J = 28.0 Hz, 24H). FTIR (ATR, Figure S9): 2956, 2932, 2866, 2239, 1727, 1610, 1445 (s), 1364, 1323, 1291, 1263 (s), 1212, 1199, 1108, 1049, 1005, 875, 858, 753 cm^−1^.*

#### 2.3.2. Synthesis of Alternating Copolymer

The alternating copolymer PIM-DMTTSBI-A50 was prepared by reaction of trimer and DMTTSBI (Figure S2). The polymerization was performed according to 2.4.1 with some minor changes. Comonomer DMTTSBI (1.11 g, 3.00 mmol) was dissolved in DMAc (10 mL), resulting in a brownish solution. The insoluble trimer (2.0 g, 3.00 mmol) was added to the solution giving a yellow suspension. The mixture was stirred for about 10 minutes before K_2_CO_3_ (0.86 g, 6.20 mmol) was added. Instantly, the flask was put in a hot oil bath (150 °C). After 1 minute, the reaction mixture turned green, bubbles evolved and the insoluble trimer dissolved completely. After 5 minutes, a yellow solid precipitated. To ensure the formation of high molecular weights of the polymer, the reaction was not precipitated in water (250 mL) before another 10 minutes. After filtration and drying in vacuum overnight at 100 °C the copolymer was further cleaned by reprecipitation from CHCl_3_ in MeOH and treatment with acetone according to the procedure described in 2.4.2. This yielded in 2.7 g polymer (4.90 mmol, 94%). Prior to further modifications, characterization was done by ^1^H-NMR, FT-IR and SEC (Table S1).

*PIM-DMTTSBI-A50: ^1^H-NMR (500 MHz, Chloroform-d, Figure S5) δ 6.82 (s, 2H), 6.68 (s, 2H), 6.45 (s, 2H), 2.37–2.05 (m, 8H), 1.73 (s, 6H), 1.42–1.19(s, 24H). FTIR (ATR, Figure S6): 2955, 2930, 2865, 2220, 1712, 1610, 1444 (s), 1364, 1323, 1290, 1263 (s), 1212, 1108, 1089, 1047, 1002, 875, 858, 753 cm^−1^.*

### 2.4. Modification of the Polymers

#### 2.4.1. Synthesis of Brominated Homopolymer and Copolymers

Brominated polymers (PIM-Br-DMTTSBI-100, PIM-Br-DMTTSBI-R50, PIM-Br-DMTTSBI-A50) were prepared in chlorobenzene (approx. 10 wt %) using BPO (1.5 wt % regarding polymer mass) as radical starter and *N*BS (8 times with respect to the amount of polymer) as bromine source at 120 °C. The addition of BPO and *N*BS caused a color change of the clear solution from yellow to orange. After 8 h the reaction mixture was poured into methanol, stirred for several hours, then filtrated and dried overnight in vacuum at 100 °C. To remove residual *N*BS, BPO and chlorobenzene the yellow solid was dissolved in chloroform and precipitated in methanol, washed with acetone and dried 24 h in vacuum at 100 °C. The synthesized polymers were characterized by means of ^1^H-, ^13^C-NMR, FTIR (ATR) and SEC (Table S1) *Yields: 88%, 85% resp. 84%.*

*PIM-Br-DMTTSBI-100: ^1^H-NMR (500 MHz, Chloroform-d, Figure S11) δ 6.87 (s, 2H), 4.04 (d, J = 137.5 Hz, 4H), 2.70–2.00 (m, 4H), 1.53–1.01 (m, 12H). ^13^C-NMR (126 MHz, CDCl_3_, Figure S13) δ 149.36, 148.84, 143.59, 142.93, 140.24, 139.81, 139.17, 138.46, 138.04, 137.64, 122.87, 111.51, 108.85, 94.99, 94.54, 94.08, 77.31, 77.05, 76.80, 58.05, 57.89, 56.08, 43.56, 43.23, 32.29, 29.57, 21.82. FTIR (ATR, Figure S12): 2956, 2866, 2239, 1719, 1611, 1578, 1435 (s), 1325, 1294, 1265 (s), 1223, 1197, 989, 865 cm^−1^.*

*PIM-Br-DMTTSBI-R50: ^1^H-NMR (500 MHz, Chloroform-d, Figure S8) δ 6.89 (s, 2H), 6.83 (s, 2H), 6.44 (s, 2H), 4.08 (d, J = 137.1 Hz, 4H), 2.70–2.01 (m, 8H), 1.54–1.09 (m, 24H). ^13^C-NMR (126 MHz, Chloroform-d, Figure S10) δ 149.73, 149.31, 146.93, 142.88, 139.24, 138.46, 122.83, 112.33, 111.51, 109.17, 94.53, 94.09, 58.81, 58.04, 56.18, 43.57, 32.29, 31.37, 29.92, 29.59, 21.88. FTIR (ATR, Figure S9): 2956, 2865, 2241, 1719, 1611, 1577, 1440 (s), 1365, 1313, 1292, 1264 (s), 1224, 1199, 1032, 1011, 873, 753 cm^−1^.*

*PIM-Br-DMTTSBI-A50: ^1^H-NMR (500 MHz, Chloroform-d, Figure S5) δ 6.86 (m, 4H), 6.45 (s, 2H), 4.41–3.66 (m, 4H), 2.69–2.09 (m, 8H), 1.59–1.10 (m, 24H). ^13^C-NMR (126 MHz, Chloroform-d, Figure S7) δ 149.73, 149.31, 146.93, 142.88, 139.24, 138.46, 122.83, 112.33, 111.51, 109.17, 94.53, 94.09, 58.81, 58.04, 56.18, 43.57, 32.29, 31.37, 29.92, 29.59, 21.88. FTIR (ATR, Figure S6): 2956, 2931, 2865, 2240, 1720, 1610, 1442 (s), 1365, 1313, 1292, 1265 (s), 1031, 1011, 997, 956, 874 cm^−1^.*

#### 2.4.2. Substitution of Bromine by Azide Group

**Figure 2:**
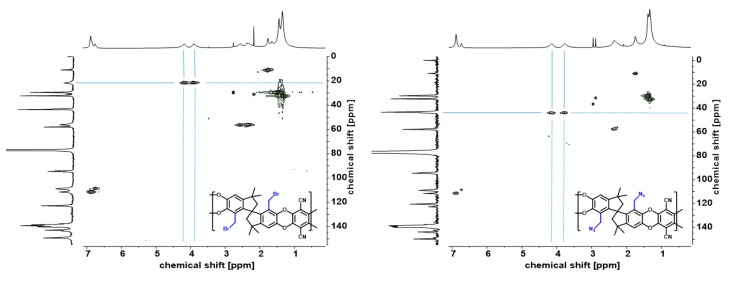
HSQC (^1^H/^13^C) spectra of brominated homopolymer (PIM-Br-DMTTSBI-100, left) and azide substituted homopolymer (AZ-PIM-100, right).

The brominated polymers (9.00 mmol) were dissolved in a mixture of THF and DMF (2:1, 0.03 mmol/mL), which led to an orange-brownish solution. Solid sodium azide (36.00 mmol) was added and the reaction mixture was heated to 60 °C. After 4 h, the azide polymers were isolated by precipitation in water and filtration. After drying in vacuum at 50 °C overnight, the polymers were reprecipitated from chloroform in methanol and dried in high vacuum (<2 × 10^−3^ mbar) at 50 °C for 48 h. This resulted in yellow solids (yields 90, 93 resp. 92%). Structural characterizations were performed by means of ^1^H-/^13^C-NMR as well as correlation of both (HSQC, Figure 2). Additionally, SEC (Table S1) for molecular weight determination and TGA (Table 1) were performed.

*AZ-PIM-100: ^1^H-NMR (500 MHz, Chloroform-d, Figure S12) δ 6.88 (s, 2H), 3.85 (d, J = 187.5 Hz, 4H), 2.34 (s, 4H), 1.49–1.08 (m, 12H). ^13^C-NMR (126 MHz, CDCl_3_, Figure S13) δ 149.77, 143.87, 140.08, 139.75, 139.09, 138.55, 137.64, 120.51, 111.53, 108.97, 94.92, 94.59, 94.19, 77.28, 77.03, 76.78, 57.87, 57.38, 44.04, 43.35, 32.23, 29.65, 29.34. FTIR (ATR, Figure S12): 2957, 2867, 2240, 2292 (s), 1727, 1674, 1613, 1577, 1436 (s), 1323, 1294, 1266 (s), 1215, 1165, 1051(s), 991, 866 cm^−1^.*

*AZ-PIM-R50: ^1^H-NMR (500 MHz, Chloroform-d, Figure S9) δ 6.88 (s, 2H), 6.82 (s, 2H), 6.43 (s, 2H), 3.79 (d, J = 184.8 Hz, 4H), 2.64–2.07 (m, 8H), 1.47–1.16 (m, 24H). ^13^C-NMR (126 MHz, CDCl_3_, Figure S10) δ 149.73, 146.97, 143.81, 140.07, 139.28, 138.49, 120.49, 112.33, 111.52, 110.57, 109.16, 94.54, 94.15, 77.30, 77.05, 76.79, 58.83, 57.90, 57.17, 43.62, 43.40, 32.19, 31.38, 29.93, 29.67. FTIR (ATR, Figure S9): 2956, 2932, 2866, 2240, 2092 (s), 1727, 1677, 1611, 1578, 1439 (s), 1315, 1291, 1264, 1213, 1109, 1047, 1012, 998, 873 cm^−1^.*

*AZ-PIM-A50: ^1^H-NMR (500 MHz, Chloroform-d, Figure S6) δ 6.83 (s, 4H), 6.44 (s, 2H), 4.05 (d, J = 184.8 Hz, 4H), 2.37–2.17 (s, 8H), 1.74–1.32 (m, 24H). ^13^C-NMR (126 MHz, CDCl_3_, Figure S7) δ 149.73, 146.97, 143.81, 140.07, 139.28, 138.49, 120.49, 112.33, 111.52, 110.57, 109.16, 94.54, 94.15, 77.30, 77.05, 76.79, 58.83, 57.90, 57.17, 43.62, 43.40, 32.19, 31.38, 29.93, 29.67. FTIR (ATR, Figure S6): 2956, 2934, 2866, 2240, 2092 (s), 1727, 1677, 1611, 1578, 1439 (s), 1365, 1315, 1291, 1264 (s), 1213, 1109, 1047, 1012, 998, 873 cm^−1^.*

### 2.5. Methods

For descriptions of equipment and evaluation methods used for NMR, FT-IR, SEC, DSC, TG-FTIR, density and gel fraction determination as well as thick film preparation and execution of the determination of the gas separation performance via time-lag method, see Appendix A.

## 3. Results and Discussion

### 3.1. Homopolymer and Copolymers

To prevent undesired involvement of the azide groups during the high temperature polymerization method (150 °C), DMTTSBI and TFTDN were polymerized first and later subjected to post-modification to get azide modified PIM structures instead of modifying the monomer. Three different polymers were prepared in order to study different degrees of functionalization as well as different distribution of the functional groups along the polymer chain. Chemical structures of the prepared polymers and their modification are shown in Figure 1. In the course of the discussion unmodified polymers are called PIM-DMTTSBI-100, -R50 and –A50. Where -100 stands for the homopolymer, -R50 for the randomly distributed copolymer and –A50 for the perfectly alternating copolymer. Furthermore, brominated polymers are named PIM-Br-DMTTSBI and azide modified polymers AZ-PIM.

PIM-DMTTSBI-100 and PIM-DMTTSBI-R50 were synthesized according to a procedure described earlier [35]. Minor modifications in reaction time and concentration were made for achieving high molecular weight polymers essential for film formation and membrane application. Subsequently, the alternating copolymer PIM-DMTTSBI-A50 was successfully prepared. Changes with regard to the concentration were made due to the different reactivity of the trimer (Figure S1) compared to TFTDN [37]. A first attempt in polymer synthesis, following the “low-temperature procedure” described by Budd and McKeown [33,34], at 55 °C in DMF and conc. of 3 mmol TFTDN/ 20 mL DMF led to a low molecular weight of the copolymer (*M*_w_ (RI): 53 kg/mol, *M*_w_ (MALS): 69 kg/mol, *M*_w_ (Visc): 57 kg/mol) which was in accordance to values achieved by Zhang et al. employing the tetrahydroxyanthracene monomer [37]. However, this was not high enough when regarding further functionalization, since the following bromination was expected to lead to partial degradation of the polymer chains [24]. The same was observed for attempts using the “high-temperature synthesis” at 150 °C in DMAc and concentration of 3 mmol TFTDN/10 mL DMAc with addition of the co-solvent diethyl benzene. Eventually, it turned out that the purity of the trimer is the limiting factor for realizing high molecular weights in polymerization. Therefore, an extensive cleaning procedure for the trimer was applied as described in 2.4.2. For polymerization, the concentration of monomers was doubled and other parameters of the “high-temperature method” were applied. These changes resulted in a very fast reaction that was terminated after 10 minutes. Molecular weight determination by means of SEC resulted in an average molecular weight (*M*_w_) of 138.7 kg/mol and a dispersity (D) of 5.9. Since the molecular weight was determined with the help of a RI detector which was calibrated with PS standard materials they are comparable with the values published by Zhang et al. [37]. It can be concluded that the changed preparation of the trimer as well as the adjusted parameters for the polymerization lead to much higher molecular weights and therefore are more convenient for the bromination, which is expected to lead to an decrease in molecular weight [24].

### 3.2. Brominated Homopolymer (PIM-Br-DMTTSBI-100) and Copolymers (PIM-Br-DMTTSBI-R50 and PIM-Br-DMTTSBI-A50)

The brominated structures were prepared via the Wohl-Ziegler reaction, a free radical substitution of hydrogen in benzyl position of the DMTTSBI unit by bromine. BPO and *N*BS were used as a radical initiator and bromine source, respectively. Reaction parameters led to reproducible degrees of 80% (±5%) bromination after 8 h. As it can be seen from Figures S5, S8 and S11, the signal of the methyl group of DMTTSBI units at 1.7 ppm is considerably reduced after bromination. Furthermore, two new signals at 4 ppm arose, which are caused by 2 benzylic protons adjacent to bromine. These two separated signals are a result of the configurational difference due to steric hindrance in DMTTSBI segments. Similar results were reported by Mei et al. for fluorenyl segments in poly (fluorine ether sulfone)s [38]. Additional signals in the aromatic region of the proton spectra can be explained by unmodified parts of the polymers. The degree of conversion, also the quantification of benzyl bromide groups was calculated by integration of the corresponding proton signals and setting them in relation, according to the number of protons that generated the signal, as described elsewhere [39]. As expected, molecular weights decreased considerably during the bromination step (see Table S1).

### 3.3. Benzyl Azide Homopolymer (AZ-PIM-100) and Copolymers (AZ-PIM-R50 and AZ-PIM-A50)

The final modification step was performed using an excess of sodium azide with respect to the benzyl bromide groups in order to receive fast and complete substitution. Moreover, as solvent a mixture of THF and DMF was used, since the brominated polymers are not soluble in DMF, but solubility of sodium azide is much higher in DMF than in THF. A ratio of 1 part of DMF and 2 parts of THF was found to be the best mixture. It is worth mentioning that during characterization of the modified polymers by SEC the determination of molecular weights by means of RI detector led to unreasonable results (Table S1). Molecular weights below the certain molecular weight, which is necessary for film formation, were measured and it is concluded that different expansion properties of the AZ-PIMs in chloroform compared to the previous polymers and especially to polystyrene standards led to these results. This is supported by a comparison of refractive index increments, dn/dc. While dn/dc-values of PIM-DMTTSBI and PIM-Br-DMTTSBI polymers are in the same order of magnitude as polystyrene (0.19–0.17, polystyrene in chloroform 0.16), AZ-PIMs differ significantly from that (0.10). This kind of deviant behavior was discussed by Bengtson et al. for modified PIMs with different poly(ethylene glycol)s and poly(propylene glycol)s [40]. 

The successful conversion of all PIM-Br-polymers to AZ-PIMs was verified by ^1^H-NMR. Additionally, in order to prove the complete conversion of the benzyl bromide groups to benzyl azide ^13^C-NMR and HSQC spectra were recorded and are shown in Figure 2.

Due to the disappearance of the signal at 21.8 ppm and the appearance of a new signal at 43.4 ppm in the ^13^C-NMR spectra, as well as the change of the coupling constant *J* of the two proton signals at around 4 ppm in ^1^H-NMR spectra from 137 to 185 Hz, the new functionality was evidenced. Furthermore, additional corroboration for a complete and successful conversion was observed by means of FTIR (ATR) (Figures S9 and S12) and TG-FTIR measurements. In the FTIR(ATR) spectrum a new strong band at 2091 cm^−1^ (asymmetric stretching vibration of R–N=N=N) arose after substitution of bromine by azide and an increase in areas in the range of 1600 to 1700 cm^−1^ (C–N stretching vibrations) was observed, indicating the successful conversion to azide groups. After substitution of bromine by azide, TG-FTIR showed a significant decline of the onset temperatures for the first step of degradation from 340 to 230 °C for copolymers and 360 to 230 °C for the homopolymer. Simultaneously recorded gas phase infrared spectra of degradation products comprised for the benzyl bromide containing polymers the typical rotational-vibrational spectrum of hydrogen bromide in the range of 2700 to 2300 cm^−1^ [24]. In contrast to that Azide-PIMs start to degrade at even lower temperatures than brominated PIMs, but the FTIR measurements did not reveal any leaving group. Figure S14 shows the TGA curve as well as the corresponding Gram-Schmidt curve. From these results, it was concluded that the azide groups are cleaved into elementary nitrogen and a nitrene radical, which then induces a crosslinking reaction similar to the observations by Wang, Eroglu and Güven [41,42]. Furthermore, TGA graphs up to 300 °C show equivalent results, independently of the atmosphere (Argon or Air, Figure S14, Table 1) with a first step of degradation that quantitatively corresponds well to the theoretical amount of nitrogen gas leaving the structure during the azide cleavage. The step height for the nitrogen release is proportional to the degree of functionalization. Increasing the temperature up to 400 °C in argon atmosphere leads to a slow but constant decrease of the sample mass, followed by a strong decrease of around 30% to 40%. In air, a more distinct weight loss between 350 and 650 °C is apparent, containing two degradation steps yielding in a residual mass of around 2% (Table 1) at 800 °C. Since this step is equal in height for both copolymers and the homopolymer, this last weight losses at high temperatures can be easily associated with the degradation of the polymer backbone. 

The crosslinking reaction between 150 and 300 °C was further investigated by DSC measurements (Figure S15, Table 1) and isoconversional kinetic analysis. In all cases a strong and irreversible exothermic peak with a maximum at around 250 °C, whose area is proportional to the degree of modification similar to step height in TGA (Figure S14, Table 1), can be observed.

For the isoconversional kinetic analysis of the three polymers, TGA in inert atmosphere was performed, using a differential method according to Friedman and an integral method according to Kissinger for evaluation. Computed *α* vs. *T* and *dα*/*dt* vs. *T* graphs for different heating rates and subsequent linear regression resulted in apparent activation energies (*E*_A_) of around 160 kJ/mol in argon. These values are in very good accordance with values published in the literature for azide-modified materials that undergo an azide cleavage [43,44,45,46]. 

**Figure 3:**
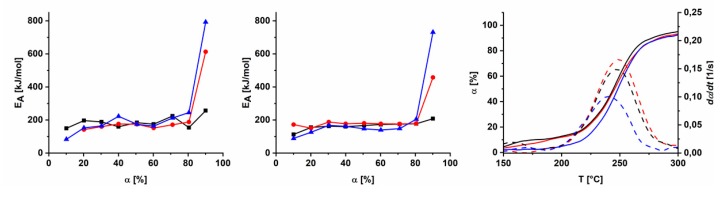
Apparent activation energy E_A_ at different conversion α of AZ-PIM-100 (black), AZ-PIM-R50 (red) and AZ-PIM-A50 (blue) according to Friedman (left) and Kissinger (middle) and for attribution of temperatures, conversion and activation energies a dα/dt-T-plot (right).

As can be seen in Figure 3, *E*_A_ increases in the range of 0.00 < α < 0.15 (150 to 200 °C), levels from 0.2 < α < 0.65 (210 to 250 °C) and subsequently starts to increase. The increase during the first interval can be explained with the initiation of the azide cleavage. The constant values up to α = 0.65 are a result of the ongoing process and the strong increase above α = 0.7 indicates the start of an additional degradation, crosslinking or rearrangement processes in the polymer samples. Hence, treatments of polymer films at different temperatures (150, 200, 250, 300 °C) within the range of the crosslinking process were performed. Using FTIR (ATR), gel fraction tests and density measurements a first series of characterizations was executed.

**Figure 4:**
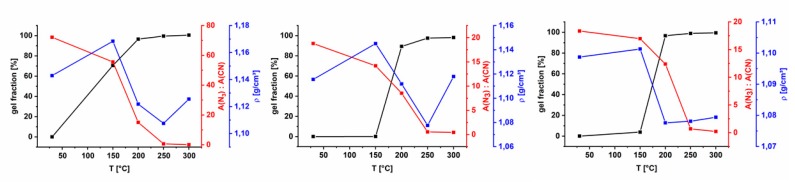
Results of gel fraction tests (black symbols), density measurements (blue symbols) and the ratio of band areas of nitrile group and azide group from FTIR (ATR) measurements (red symbols) at different crosslinking temperatures of AZ-PIM-100 (left), AZ-PIM-R50 (middle) and AZ-PIM-A50 (right).

As shown in Figure 4, the increase in gel fraction of the three polymers is different. This is obviously a result of different degrees of functionalization and of a different distribution of the azide groups among the polymer backbone. In case of AZ-PIM-R50, the decrease of the ratio of azide versus nitrile groups at 150 °C is more distinct, compared to AZ-PIM-A50. However, the former polymer results in a completely soluble material, whereas a gel fraction of about 4% was determined in the case of AZ-PIM-A50. Additionally, at 200 °C a gel fraction of 97% was already obtained for AZ-PIM-A50, while only 89% for AZ-PIM-R50 was achieved. Consequently, the distribution of azide groups plays an important role for the solubility and therewith for the crosslinking. The results from density measurements, which are also shown in Figure 4, make clear that the different treatment temperatures lead to different processes, which in turn lead to different film morphologies of the treated polymers. In all cases a slight increase in density of the polymer films after the treatment at 150 °C was observed, which represents the start of the azide cleavage and the formation of a small quantity of linkages. This can be correlated with the results of the kinetic data analysis (TGA) for the first interval, with a slow increase of *E*_A_ (0.00 < α < 0.15). An equal behavior for all materials was observed for 200 and 250 °C. A drop of density below the values of the untreated polymers imply the formation of linkages, which apparently provide bigger distances of the polymer chains. In addition, the release of large amounts of nitrogen due to the azide degradation is expected to act as a blowing agent. This was concluded from the comparison of density values of temperature treatments at 150, 200 and 250 °C. Even though the crosslinking mechanism is expected to be the same at all three temperatures, density after treatment at 150 °C slightly increased whereas at 200 and 250 °C densities decreased significantly. The only difference between treatments at 200 and 250 °C is the amount of nitrogen released during the process. However, an exception in the case of density changes is AZ-PIM-A50. No significant change in the range of 200 to 250 °C was found. Furthermore, at 250 °C it seems to lead to a minimum in density values, which can be correlated with the peak temperature of the exothermic effect in DSC and the maximum conversion rate *dα*/*dt* of the process (Figure 3). Since density values at 300 °C increased again, it can be concluded that other reactions take over at this temperature forming other linkages or starting partial degradation of the network. This again is in accordance with isoconversional kinetic studies described earlier. While the values of *E*_A_ are nearly constant up to 250 °C and thus similar processes are proceeding, *E*_A_ above 250 °C drastically increases, which implies a significant change in reactions that occur during the process.

**Figure 5:**
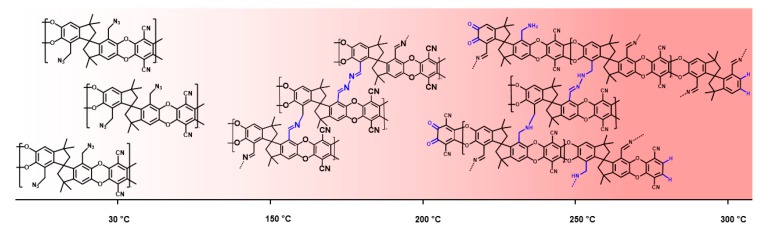
Expected structures of AZ-PIMs at different temperatures.

However, since the structures of the formed networks are of major interest, FTIR (ATR, Figure S16 left) and solid-state NMR (CP-MAS, Figure S16 right) studies were conducted. IR-spectra depict clearly the on-going cross-linkage by diminishing of the azide band at 2090 cm^−1^. Suggested structures after the different temperature treatments are provided in Figure 5. From FT-IR spectra (at 3000–2800 cm^−1^) can be concluded that no reaction of the products of the azide cleavage took place with -CH_3_, -CH_2_ (TTSBI unit) or residual unmodified –CH_3_ (bound to aromatic ring of TTSBI). These findings were corroborated by analyzing relevant signals of the polymer backbone in IR, such as the –C–O–C– (1050 cm^−1^), –C–C–H (1436 cm^−1^) and –C–C– (1266 cm^−1^) vibration. This was further supported by solid state NMR. Therefore, it is expected that the crosslinking at lower temperatures (up to 200 °C) in a first step is combined with the formation of imine functionalities after the azide cleavage and a further reaction of these functionalities with other imine groups or products of the azide cleavage as described in [41,46]. Whether this reaction takes place either intra- or intermolecular could not be verified. As can be seen from solid-state NMR spectra (Figure S16, right) of the differently treated polymers, the three signals in the range of 20 to 60 ppm remained nearly constant. Small changes were observed for the signal at 43 ppm that is attributed to the cleavage of the azide modification. At higher temperatures additionally the signal at around 59 ppm slightly decreased. Since that change was observed at temperatures higher than 250 °C it can be correlated with the partial degradation of the polymer backbone or further reactions; however, changes in structure that are not directly linked with reactions of the remaining nitrogen radical after azide cleavage. The degradation of the polymer backbone became conclusive because a new signal at 250 and 300 °C appeared in CP-MAS spectra at around 184 ppm, which can be assigned to carbonyl functionalities. Additionally, the corresponding FTIR spectra showed a decrease of C–O–C stretching vibrations at 1050 cm^−1^ and an increase in C=O stretching vibrations at around 1700 cm^−1^ (Figure S16, left). Therefore, it is expected that the ether bonds of the polymers are cleaved at these temperatures and form quinone functionalities. Temperatures above 250 °C led to the disappearance of the singlet at 159 ppm, which is attributed to imine functionalities, and a further increase of the signal at 184 ppm was apparent. The corresponding FTIR spectra show a significant increase in areas at around 1700–1550 cm^−1^ and new bands in the range of 3100–3600 cm^−1^ arose. These observations finally led to the assumption that besides the formation of imine functionalities at high temperatures (250 to 300 °C) primary and secondary amines are formed (Figure 5). Hence, at these temperatures potentially further conversions due to addition reactions or hydrolysis with degradation products or rearrangements of the imine functionalities may occur.

### 3.4. Gas Separation Performance

**Figure 6:**
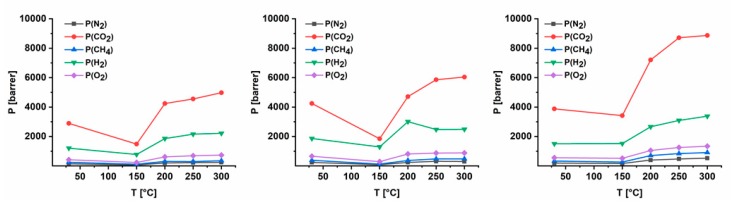
Permeability coefficients of AZ-PIM-100 (left), AZ-PIM-R50 (middle) and AZ-PIM-A50 (right) measured approximately 20 h after temperature treatments.

The gas separation characteristics were studied by means of solvent cast thick films prepared from azide containing polymers that were treated at 150, 200, 250 and 300 °C. The permeability, solubility and diffusivity coefficients of CO_2_, CH_4_, N_2_, O_2_ and H_2_ as well as corresponding selectivity pairs are provided in Tables S2–S4. Figure 6 shows permeability coefficients of the three synthesized AZ-PIM polymers depending on crosslinking temperature. It can be seen that the values of permeability of the untreated azide modified homopolymer (AZ-PIM-100) are about 30% lower than the values of the copolymers due to a higher degree of modification and the associated occupation of free volume elements. After treatment at 150 °C, a slight decrease in permeability occurred, whereas the permeability of each gas increased drastically at temperatures higher than 200 °C. This behavior is in correlation with the densities of the films and proportional to the amount of released nitrogen. As mentioned earlier it is expected that nitrogen act as expanding agent. Due to their size and accordingly to their low diffusion coefficients, nitrogen molecules leave the polymer matrix comparatively slow. Additionally, because of the high degradation rates of the azide groups at high temperature (>200 °C) the concentration of nitrogen molecules escalates within the free volume elements, so that the polymer chains are pushed apart from each other. Therefore, significant differences in permeability changes of the three materials became obvious. The permeability values of the homopolymer (AZ-PIM-100) at first halved at 150 °C and then nearly doubled up to 300 °C compared to the values of the untreated film. In case of copolymer AZ-PIM-R50 the value for 150 °C also halved and increased 1.5 times compared to the starting value. The values for AZ-PIM-A50 at 150 °C were nearly constant compared to the untreated material and at 300 °C even more than doubled.

However, the importance of nitrile groups for high permeability coefficients and CO_2_ affinity of PIM-1 is often neglected with regard to its gas performance. As can be seen from literature, even after simple modification of this group (for example to carboxylic acid, amino groups or thioamide functionalities) permeability coefficients drop [47,48]. More precisely, solubility coefficients decrease slightly or remain constant while diffusion coefficients decrease drastically because these new functional groups occupy residual free volume elements. The same behavior was found in literature crosslinking PIM-1 by use of the nitrile group as point of coupling [26,27,31,32]. Therefore, in the present work spirobisindane units of PIM-1 were modified for crosslinking, in order to retain the advantageous properties that derive from the nitrile group. While in the above-mentioned articles the often-described tradeoff between permeability and selectivity was observed, in case of azide modified PIMs permeability increased during cross linking and selectivity remained nearly constant (Table S2). Reason for these differences is supposedly the already described effect of the nitrogen release that mainly results in an abrupt rise in diffusivity in case of all tested gases (Table S4). As an example of that, diffusivity coefficients of CH_4_ and CO_2_ are shown in Table 2. It becomes clear that in all cases at temperatures higher than 200 °C the diffusivity coefficient increases significantly and in case of AZ-PIM-A50 even doubled. In addition, the formation of new functionalities like imine groups (Figure 5) and remaining azide groups lead to a slight increase in solubility coefficients (Table S3). These functionalities may act as Lewis base and hence lead to an increase in affinity especially to CO_2_ and CH_4_ (Table 2). 

**Figure 7:**
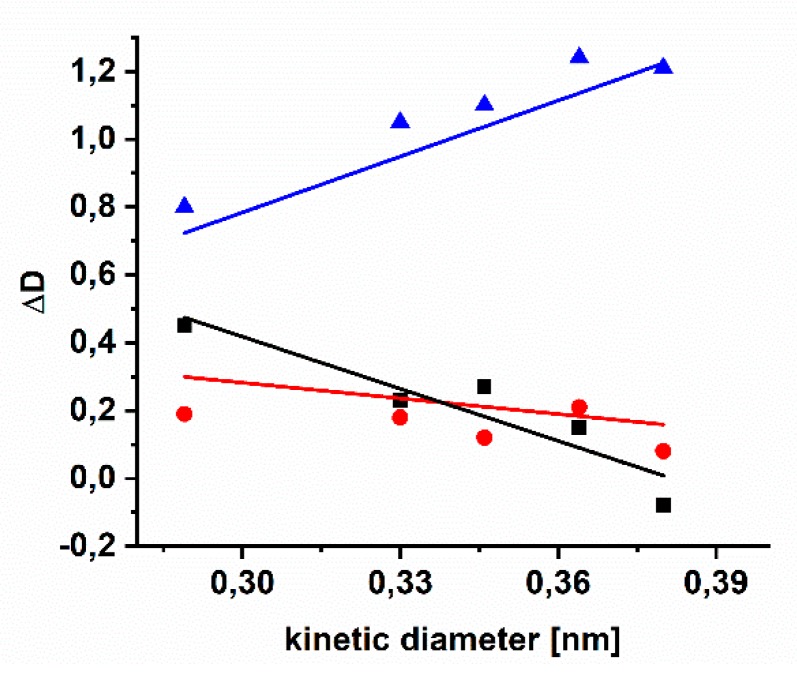
Change of diffusivity coefficient of different gases depending on their kinetic diameter (ΔD = (D_250 °C_ − D_30 °C_)/D_30 °C_) (AZ-PIM-100 (black squares), AZ-PIM-R50 (red circles), AZ-PIM-A50 (blue triangles)).

As can be seen in Figure 7 for AZ-PIM-100 there is nearly no change in diffusivity of CH_4_ or N_2_, while diffusivity in case of the smaller gases changes significantly. This effect is less accented in case of AZ-PIM-R50 due to a lower concentration of crosslinking groups. However that means new smaller free volume elements are generated in case of these two polymers during crosslinking. In case of AZ-PIM-A50, a contrary behavior was observed. All gases show a more distinct increase in diffusivity coefficients, especially for gases with large kinetic diameters like N_2_ and CH_4_. It can be concluded that larger free volume elements are generated in this material. The reason for that might be the uniform distribution of crosslinking groups. In this copolymer, mostly the PIM-1 backbone generates the free volume before crosslinking. Free volume elements in this state are partially occupied by azide groups, leading to lower permeability and higher selectivity than PIM-1. During the crosslinking, nitrogen is released, new covalent bonds are formed and the already existing free volume elements can be interconnected due to the cleavage of azide groups. That leads to a jump in diffusivity as a result of the generation of a larger overall accessible free volume. In case of AZ-PIM-100 this is not possible due to a higher degree of modification and a higher number of linkages. In case of AZ-PIM-R50 the unpredictable and not adjustable distribution of the DMTTSBI monomer during polymerization might lead to longer PIM-1 like and DMTTSBI rich segments. Subsequently, the effect of the crosslinking cannot be as prominent as in case of AZ-PIM-A50 or AZ-PIM-100.

### 3.5. Aging of Cross Linked Azide PIMs

**Figure 8:**
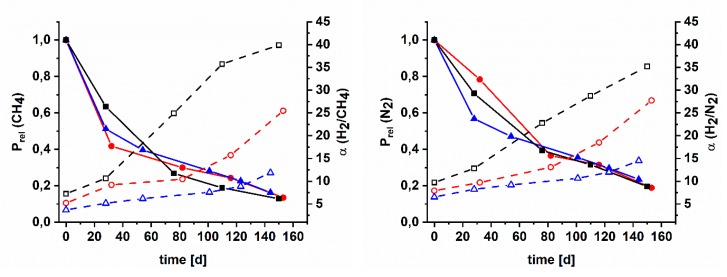
Relative permeability of CH_4_ (left) and N_2_ (right) (solid lines) versus time of AZ-PIM-100 (black squares), AZ-PIM-R50 (red circles) and AZ-PIM-A50 (blue triangles) and change of H_2_/CH_4_ and H_2_/N_2_ selectivity (dashed lines) during compaction.

Since polymers of intrinsic microporosity usually suffer from an intense physical aging, temperature treated films were tested for their aging behavior. They were stored in a desiccator for about five months and during this time, the gas transport properties were measured several times. As can be seen in Figures S17, S19 and S21 the relative permeability of the crosslinked membranes decreased with time. As a result of this compaction, on account of the collapse of large free volume elements and the reduction of distances between polymer chains, the size sieving behavior is improved. It results in an increase in selectivity, noteworthy after treatments at 200 °C and higher (Figures S18, S20 and S22). Presented in Robeson plots, it can be seen that the crosslinking by nitrene reaction had shifted the position of the AZ-PIMs towards the Robeson upper bound, without a significant lost in selectivity compared to the boost in permeability (Figure S23). During aging time especially the selectivity of H_2_ over CH_4_ as well as H_2_ over N_2_ increased (Figure 8) and significantly surpassed the Robeson upper bound. A similar behavior but not that distinct was observed for the selectivity of CO_2_ over CH_4_ as well as CO_2_ over N_2_. In case of AZ-PIM-100, after treatment at 250 °C, the selectivity of H_2_/CH_4_ quintupled from 7.2 to 39.9 during aging and H_2_/N_2_ quadrupled from 9.7 to 35.2, whereas CO_2_ selectivity doubled from 15.3 to 31.9. Similar observations were made for both copolymers; selectivity of H_2_/CH_4_ rose from 3.7 to 11.9, H_2_/N_2_ from 6.5 to 14.5 and CO_2_/CH_4_ from 10.4 to 19.5 in case of AZ-PIM-A50 (AZ-PIM-R50: H_2_/CH_4_ from 5.2 to 25.5, H_2_/N_2_ from 7.9 to 27.7 and CO_2_/CH_4_ from 12.3 to 24.6). However, the selectivity of the copolymers of all measured gas pairs was lower than the one of AZ-PIM-100. This led to the conclusion that due to a larger amount of curable functionalities the formation of smaller free volume elements is leading to an enhanced sieving effect. Copolymers AZ-PIM-A50 and AZ-PIM-R50 contain 50% of PIM-1 segments distributed differently in both copolymers. These PIM-1 segments are unperturbed by the crosslinking reaction and age differently. 

The absolute permeability of AZ-PIM-A50, however, was much higher over the whole period of time. Bernardo and co-workers proposed an approach for the quantification of the physical aging rate of gas permeability (β_P_) [12]. It claims that the trend of permeability on time presented in a log-log scale is nearly linear. Thus, the slope of a linear fit is equal to the permeability aging rate referred to the size of gas molecule used for the calculation. Furthermore, a graph of these aging rates (β_P_) of different gases versus molecular diameter demonstrates quantitative differences in aging rates of different polymers and should be helpful for the interpretation of the aging behavior. Therefore, graphs were prepared for the tested gases and the various treatment temperatures (Figure S24). Depending on the temperature treatment, the aging behavior changes remarkably and clearly depends on the degree of functionalization. Regarding untreated AZ-PIMs by the β_P_ versus kinetic diameter graph (Figure S24-A), it was found that the compaction or physical aging, respectively, of the modified homopolymer affects small free volume elements to the same extent as larger ones; the slope is 0. Consequently, constant selectivity values result over the period of aging. In case of the copolymers, the effect of aging on larger free volume elements is much more distinct. The decrease of permeability for large gas molecules like CH_4_ is more significant than for small molecules like H_2_, therefore the resulting aging rates (β_P_) are nearly two times higher in case of CH_4_ compared to H_2_. Additionally, it can be seen that aging rates of AZ-PIM-R50 are higher than with AZ-PIM-A50. The membranes that were treated at 150 °C show a similar trend. While the different free volume elements of the modified homopolymer AZ-PIM-100 after this treatment are affected quite similar, the aging rates of AZ-PIM-A50 do not change compared to the untreated material. The aging rates of AZ-PIM-R50 significantly decrease. These changes follow very well the trend of the measured density of the temperature treated materials. A higher density means lower aging, which in fact is reasonable, since physical aging is a kind of compaction. Subsequently, at temperature treatment of 200 °C and higher, a significant change in aging rates is apparent, again according to the measured density of the polymers. Aging rates of AZ-PIM-100 show a remarkable change. While aging rates of H_2_ only double up to 300 °C, the rates for CH_4_ after 250 and 300 °C treatments are about fifteen times higher. According to that, the large free volume elements that were formed on account of the crosslinking reaction, collapse much faster than the smaller ones and as well faster than comparable free volume elements within the copolymers. That means that not only the absolute values of the aging rates demonstrate quantitative differences, but also the slope of the β_P_ versus kinetic diameter corresponds to an intrinsic material behavior. As can be seen in Figure S24, this slope is nearly the same for the two copolymers in all temperature treatments above 200 °C. Accordingly, it can be concluded that the effect of the compaction on the free volume distribution is nearly comparable in both polymers, but due to the formation of larger pores or free volume elements, respectively, the aging rates of AZ-PIM-A50 are lower. That is in accordance with published results of Bernardo and co-workers, who presented aging rates of PIM-1 treated at different temperatures [12]. They claimed that the aging rates of PIM-1 treated at 75 °C are higher than with PIM-1 treated at 125 °C, but the slope of both linear fits, meaning the effect of compaction on the free volume distribution was nearly identical.

From these results it is concluded that the distribution and concentration of the curable functionalities as well as the temperature treatment have a significant effect on the aging behavior of these three polymers under examination. On one side, a high degree of functionalization, combined with a significant degree of crosslinking, leads to a polymer that is very different from the untreated polymer on regard to physical aging and the formation of a tight network. The membranes show lower permeability but the resulting increase in sieving boosts the selectivity especially for small molecules, such as hydrogen. On the other side, in case of the copolymers, the process of compaction resp. physical aging generally is the same; small and large free volume elements are affected to the same extent. However, due to the different distribution of azide groups and the accompanied formation of differently sized free volume elements, the absolute aging rates of AZ-PIM-A50 are lower than the ones of AZ-PIM-R50. AZ-PIM-A50 forms membranes, which at any time showed higher absolute permeability than the ones from AZ-PIM-R50, but due to a less effective sieving showed lower selectivity.

## 5. Conclusions

Azide modified polymers of intrinsic microporosity with different distribution of functionalities and different degrees of modification were successfully synthesized by post modification of methylated PIM-1. Another pathway of the preparation of perfectly alternating copolymers was established. The first step of modification was performed as reported by [24]. The substitution of bromine with azide in an S_N_2 mechanism was performed quantitatively and the structures were verified by ^1^H-, ^13^C-NMR as well as HSQC, SEC and FTIR (ATR) measurements. Additional thermal characterizations with TG-FTIR, DSC and kinetic analyses were applied for investigations of the occurring crosslinking reaction at increased temperatures by cleavage of azide groups. Possible structures of the resulting networks were suggested according to solid state NMR (CP-MAS) and FTIR (ATR). Studies of the gas separation performance by means of time lag experiments revealed significant differences in permeability and selectivity depending on the degree of modification of the polymers as well as on the distribution of the modification. After temperature treatments the often-observed tradeoff between permeability and selectivity was compensated by an increase in solubility as well as in diffusivity coefficients. As a result of that boost in permeability all membranes showed a performance close to the Robeson upper bound or above. Aging studies with the prepared membranes over approx. five months revealed that not only the formation of free volume elements is affected by this kind of crosslinking, but also the aging behavior. It was found that high degrees of functionalization lead to a strongly enhanced sieving effect, which especially for small molecules like H_2_ led to high selectivity. A homogeneous distribution of the curable functionalities in the copolymer PIM-AZ-A50 reduced the physical aging of the membranes.

Since azide groups are predestinated for azide-alkyne-cycloaddition reactions (e.g., generation of triazole functionalities), versatile possibilities of changing properties like hydrophilicity or carbon dioxide affinity, offer a next step regarding the investigation of these kind of AZ-PIMs. 

## Figures and Tables

**Table 1:** Results of thermal characterization of AZ-PIMs.

Polymer		*DSC*		*TGA*				
		*T*_Onset_ [°C] ^a^	*T*_Peak_ [°C] ^a^	*T*_Onset_/*T*_Inflection_ [°C] ^b^	Weight Loss [%] ^b^	Weight Loss [%] ^c^	Weight Loss [%] ^d^	Residual Mass [%]
AZ-PIM-100	*Argon*	225	257	223/243	9.4	29.3		60.5
	*Air*			225/245	9.2	24.5	65.2	1.0
AZ-PIM-R50	*Argon*	224	258	224/246	5.1	32.6		61.5
	*Air*			224/245	4.9	23.9	69.6	1.5
AZ-PIM-A50	*Argon*	224	258	224/245	5.0	39.4		53.8
	*Air*			226/246	4.7	27.8	65.1	1.7

^a^: temperatures of the nonreversible exothermic peak of the first heating cycle. ^b^: temperatures and weight loss of the first step of degradation; azide cleavage. ^c^: step height of the second step of degradation. ^d^: step height of the third step of degradation (only apparent in air).

**Table 2:** Permeability P (Barrer), diffusivity D (×10^−6^ cm^2^/s) and solubility S (cm^3^/cm^3^ cmHg) coefficients of CH_4_ and CO_2_ after thermal treatment of azide modified PIMs.

Polymer		*T* [°C]				
		30	150	200	250	300
AZ-PIM-100	P(CH_4_)	245	116	308	298	351
	P(CO_2_)	2894	1483	4239	4551	4977
	D(CH_4_)	0.20	0.10	0.19	0.17	0.20
	D(CO_2_)	0.50	0.27	0.60	0.59	0.64
	S(CH_4_)	0.13	0.12	0.16	0.18	0.18
	S(CO_2_)	0.58	0.56	0.71	0.78	0.78
AZ-PIM-R50	P(CH_4_)	384	126	368	476	479
	P(CO_2_)	4246	1846	4709	5860	6042
	D(CH_4_)	0.23	0.11	0.22	0.25	0.26
	D(CO_2_)	0.60	0.31	0.62	0.71	0.76
	S(CH_4_)	0.17	0.11	0.17	0.20	0.19
	S(CO_2_)	0.71	0.61	0.76	0.83	0.80
AZ-PIM-A50	P(CH_4_)	331	264	704	839	911
	P(CO_2_)	3879	3422	7206	8712	8868
	D(CH_4_)	0.23	0.18	0.44	0.50	0.49
	D(CO_2_)	0.60	0.53	1.07	1.24	1.23
	S(CH_4_)	0.15	0.15	0.16	0.17	0.19
	S(CO_2_)	0.64	0.65	0.67	0.70	0.72

## Supplementary Materials

The supplementary materials are available online at https://www.mdpi.com/2073-4360/11/8/1241/s1.

## Appendix A

### Methods

Size exclusion chromatography (SEC) was performed at room temperature in chloroform using a column combination [pre-column-SDV, SDV-100 Å, SDV-1000 Å, SDV-10000 Å, SDV-100000 Å] with 5 µm particle size at a flow rate of 1 mL/min, supplied by PSS Polymer Standards Service GmbH (Germany). A combination of refractive index (RI), multi angle laser light scattering (MALS) and viscosity (Visc) detector was used for the determination of the average molecular weight (*M*_w_) and dispersity (D). Polystyrene standards of different molecular weights were applied for calibration of the RI detector as well as for viscosity detector. Prior to molecular weight calculation by means of the MALS detector, the refractive index increment (*dn*/*dc*) had to be determined. Since the signal area of the RI detector is depending on concentration, it was possible by measuring five different concentrations of a polymer sample to calculate the slope of a linear fit of concentration versus signal area, the refractive index increment (*dn*/*dc*).

The synthesized materials were characterized by ^1^H-, ^19^F-and ^13^C-NMR spectroscopy employing a Bruker AVIIIHD spectrometer at 500 MHz, using deuterated *N,N*-dimethylformamide (DMF-*d*7), chloroform (CDCl_3_) or dichloromethane (CD_2_Cl_2_) as the solvent.

^1^H/^13^C-heteronuclear single quantum correlation (HSQC) was performed using a vendor supplied HSQC experiment with pulse sequence *“hsqcedetgp”* with H-1/X correlation via double inept transfer and with decoupling during acquisition (parameters: TD 1024 points, 256 rows, 16 scans, D1 1.5 s). Cross-linked materials were characterized by Cross-Polarization Magic Angle Spinning (CP/MAS) NMR spectroscopy measurements for the investigation of the thermally treated insoluble polymer films.

Differential scanning calorimetry (DSC) was performed with a differential scanning calorimeter DSC1 (Mettler-Toledo, Giessen, Germany) in a temperature range between 30 and 350 °C under a nitrogen atmosphere (60 mL/min) and at a heating rate of 10 K/min to study the thermally induced transitions of polymers. Samples of around 5 mg were heated three times. At first from 30 to 150 °C in order to remove impurities and the thermo-mechanical history of the materials. Afterwards, the samples were heated from 100 to 280 °C, cooled down to 100 °C and heated again to 350 °C.

FTIR spectra were collected on an ALPHA FTIR spectrometer (Bruker, Bremen, Germany) in attenuated total reflectance (ATR-diamond crystal) mode. The measurements were done in a spectral range of 400–4000 cm^−1^ with a resolution of 4 cm^−1^ and 64 scans.

TGA-FTIR measurements were performed on a TGA-DSC2 (Mettler Toledo, Germany) coupled with an FT-IR spectrometer Nicolet iS50 (Thermo Scientific, Germany). The samples (10–15 mg) were heated at 5 K/min from 30 to 800 °C in an Argon atmosphere or in synthetic air (O_2_/N_2_ 20/80) with a gas flow of 20 mL/min. Gas phase spectra were collected in a spectral range of 400–4000 cm^−1^, with a resolution of 4 cm^−1^ and an average of 8 scans.

Kinetic analysis, which were carried out with the help of TGA experiments in the range of 110 and 340 °C, were performed in agreement with recommendations developed by the Kinetics Committee of the International Confederation for Thermal Analysis and Calorimetry (ICTAC) [49]. According to that sample masses of 5 mg were used for each measurement using an argon gas flow of 20 mL/min. Each sample was measured with 5 different heating rates (1, 3, 5, 7.5, 10 K/min). Normalized integral curves were derived using the usual procedure and the degree of conversion of the mass loss was defined as (Equation (A1)):

(A1) $$\alpha \;=\;\frac{m_{0\;}-\;m}{m_{0}{\;-\;m}_{end}}$$

where *m* is the mass corresponding to a temperature *T*, *m_0_* is the initial mass and *m*_*end*_ is the mass of the substance at the end of the process. The rate of mass loss (*dα*/*dt*) was also calculated and can be described by following general equation for solid-state reactions (Equation (A2)):

(A2) $$\frac{d\alpha }{dt}\;=\;A\;\exp\left(\frac{-E}{RT}\right)f(\alpha )$$

*A* is the Arrhenius pre-exponential factor, *R* the gas constant (8.314 J/mol·K), *E* the activation energy, *α* the degree of conversion, *T* the process temperature and *f(**α)* accounts for the reaction rate dependence on *α*. Equation (A2) is a general expression that describes the relationship between the reaction rate, reacted fraction and temperature independently of the thermal pathway used for the obtainment of the experimental data. In the case that experimental data were recorded at constant heating rate *β = dT*/*dt*, Equation (A2) can be written as follows:

(A3) $$\frac{d\alpha }{dt}\;=\;\frac{A}{\beta }\;\exp\left(\frac{-E}{RT}\right)f(\alpha )$$

Isoconversional methods (model-free methods) are used to determine the activation energy as a function of reacted fraction without any previous assumption on kinetic model or the reaction mechanism. The Friedman isoconversional method is a widely used differential method that provides accurate values of activation energies even if the activation energy is a function of the reacted fraction. Equation (A3) can be written in logarithmic form [50]:

(A4) $$\ln\left(\frac{d\alpha }{dt}\right)\;=\;\ln\left(A\;f(\alpha )\right)-\frac{E}{RT}$$

The activation energy at a constant degree of conversion *α* can be determined from the slope of the plot of the left hand side of Equation (A4) against the inverse of the temperature *T*.

Because of its easy use the Kissinger method has been applied for determining the activation energies more extensively than many other multiple-heating rate methods. In the present work, this integral isoconversional method was used solely for verification of the results obtained with the help of the Friedman method. The estimation of activation energies in the case of the Kissinger method follows Equation (A5):

(A5) $$\ln\left(\frac{\beta }{T^{1.92}}\right)\;=\;\ln\left(-\frac{AR}{E}f\text{’}(\alpha )\right)-\frac{E}{RT}$$

In the Kissinger method, the left hand side of Equation (A5) is plotted against 1/*T* giving rise to a straight line whose slope yields the activation energy.

The gel-content of the membranes was determined by soaking the membranes in CHCl_3_ as an appropriate solvent for the materials. The films were soaked in the solvent for 72 h. They were weighed before the solvent treatment (*w*_1_) and after drying (*w*_2_) at 100 °C in high vacuum (<0.002 mbar). The gel-fraction was calculated via the following equation (Equation (A6)):

(A6) $$\mathrm{gel}\;\mathrm{fraction}\;\left(\%\right)\;=\;\frac{w_{2}}{w_{1}}\times \;100$$

Thick polymer films were prepared by solution casting from chloroform solution (3 wt %). After dissolution, the polymer solution was filtered and poured into a levelled Teflon® mold. The mold was covered and the solvent was evaporated by a slow N_2_ gas stream over 24 h. Subsequently, drying at 100 °C (60 °C for azide modification) in vacuum (2 × 10^−3^ mbar) for about 48 h led to films of 70 to 90 µm thickness. A digital micrometer (Deltascope MP2C) was used for the determination of the thicknesses of membranes. To study the effect of thermal crosslinking of the azide modified PIM’s, thick films were treated at different temperatures (150, 200, 250, 300 °C) for 30 minutes in a tubular oven (FRH-100/520/1250, Linn High Term GmbH, Eschenfelde, Germany) in nitrogen atmosphere (150 mL/min). The resulting mechanically stable, freestanding films were tested several times over a period of about 6 months with the help of gas permeation measurements (“time-lag”) in order to study the effect of aging at different degrees of crosslinking. Meanwhile the samples were stored in a desiccator.

The density of the membranes was determined using a Mettler Toledo XP105 balance (Mettler Toledo, Germany) equipped with a density determination kit. The samples were weighed in air and FLUORINERT FC-77 (3M, Neuss, Germany) was used as the auxiliary fluid to prevent liquid adsorption on the PIMs. The density was calculated using the following equation (Equation (A7)):

(A7) $$\rho_{\mathrm{membrane}}=\frac{w_{air}}{w_{air}{-w}_{liq}}{\times \rho }_{liq}$$

With *ρ*_*membrane*_ being the membrane density (g/cm^3^), *w*_air_ and *w*_liq_ being the weight of the membrane in air and FC-77 (g), respectively, and *ρ*_liq_ being the density of FC-77 (g/cm^3^).

Gas permeability was measured by a “time-lag” experimental facility developed in-house using constant volume variable pressure approach for permeability and diffusion coefficients determination [51]. The facility has already been discussed elsewhere [4,52]. The single gas permeability *P* of H_2_, N_2_, O_2_, CO_2_, and CH_4_ was determined at 30 °C and a feed pressure of 1000 mbar. Each measurement was repeated 3 times. Each sample was evacuated in the “time-lag” experimental facility for several hours prior to measurement to remove traces of gases and residual solvent. The solution–diffusion transport model was applied [53] The permeability (*P*), diffusivity (*D*), solubility (S) coefficients and ideal selectivity (α) for gases *i* and *j* were determined under steady state conditions by the following equations (Equation (A8)–(A10)) [54]:

(A8) $$P\;=\;D\;\times \;S\;=\;\frac{V_{p}{l(p}_{p2}\;{-\;p}_{p1})}{ART\Delta {t\;(p}_{f}\;{-\;(p}_{p2}{\;+\;p}_{p1})\;/2)}$$

(A9) $$D=\frac{l^{2}}{6\theta }$$

(A10) $$\alpha_{i/j}=\frac{P_{i}}{P_{j}}=\frac{D_{i}S_{i}}{D_{j}S_{j}}$$

where *V_p_* is the constant permeate volume, *R* the universal gas constant, *l* the thickness, *A* the effective area of membrane, Δ*t* the time for permeate pressure to increase from *p*_*p*1_ to *p*_*p*2_, *p_f_* is the feed pressure. *θ* is the measured time lag in permeation.
