# Supplementary material for: Thermal Cross Linking of Novel Azide Modified Polymers of Intrinsic Microporosity—Effect of Distribution and the Gas Separation Performance
Source: Polymers (Basel). 2019 Jul 26;11(8):1241. doi: 10.3390/polym11081241 (PMC6723633; doi:10.3390/polym11081241)
Supplement: Supplementary file 1 [file polymers-11-01241-s001.pdf]

Supplementary Materials

Results and Discussion

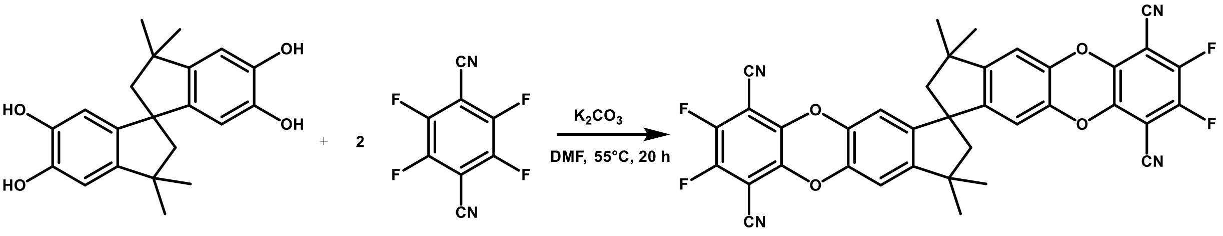

Figure S1. Synthesis of Trimer

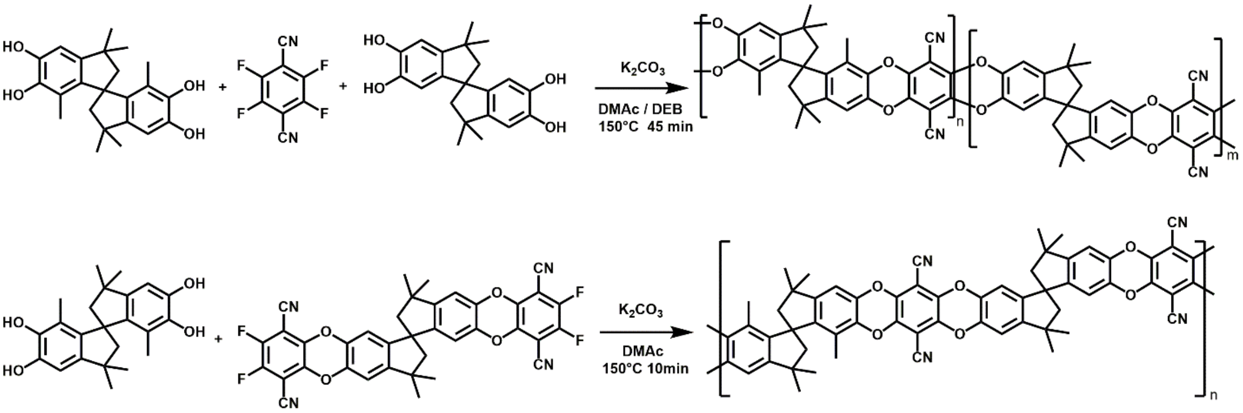

Figure S2. Preparation of the different precursor polymers for further modifications

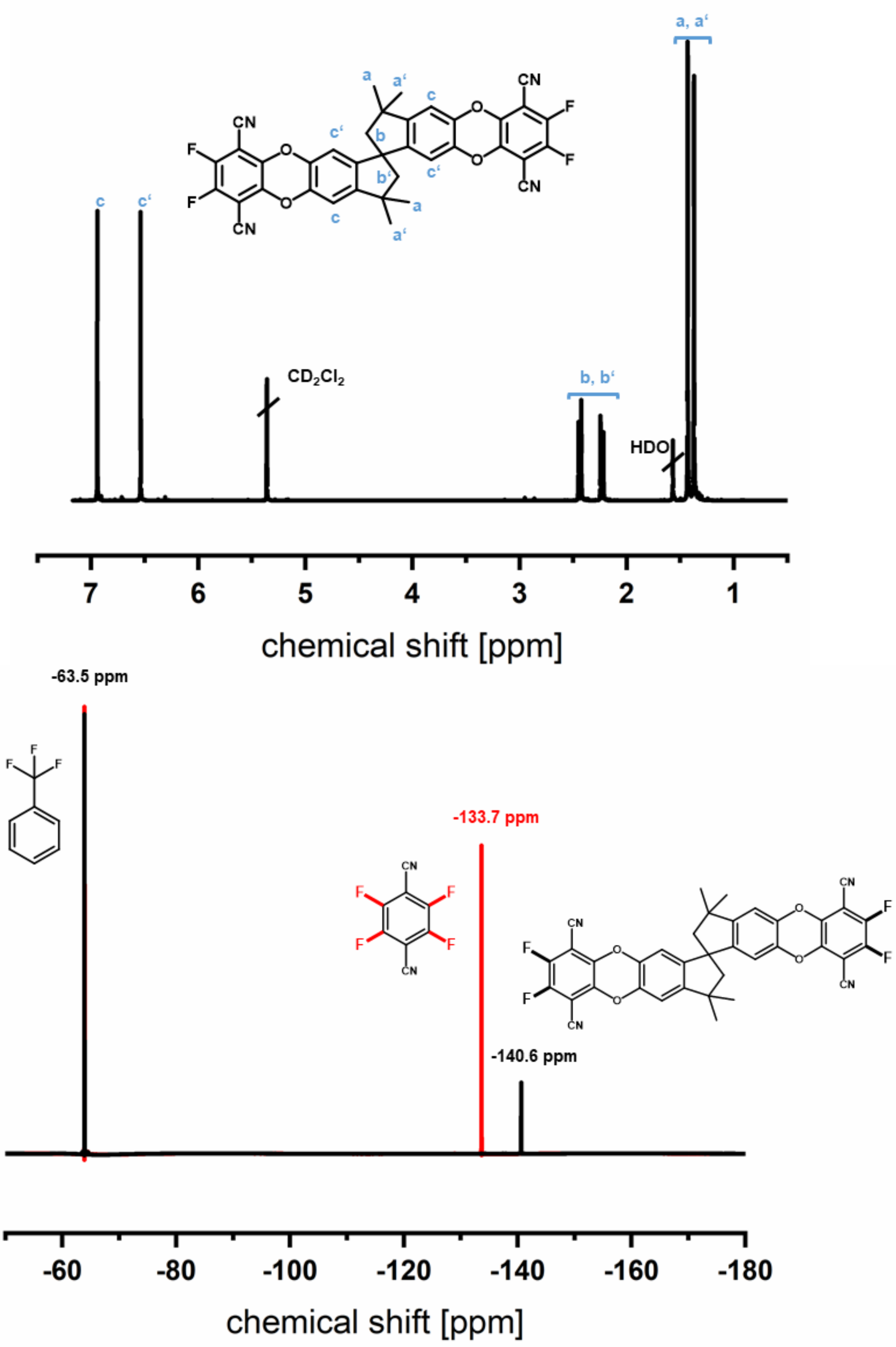

Figure S3. ^{1}H- (CD_2Cl_2) and ^{19}F-NMR (DMF-d7) spectra of synthesized trimer for preparation of an alternating copolymer

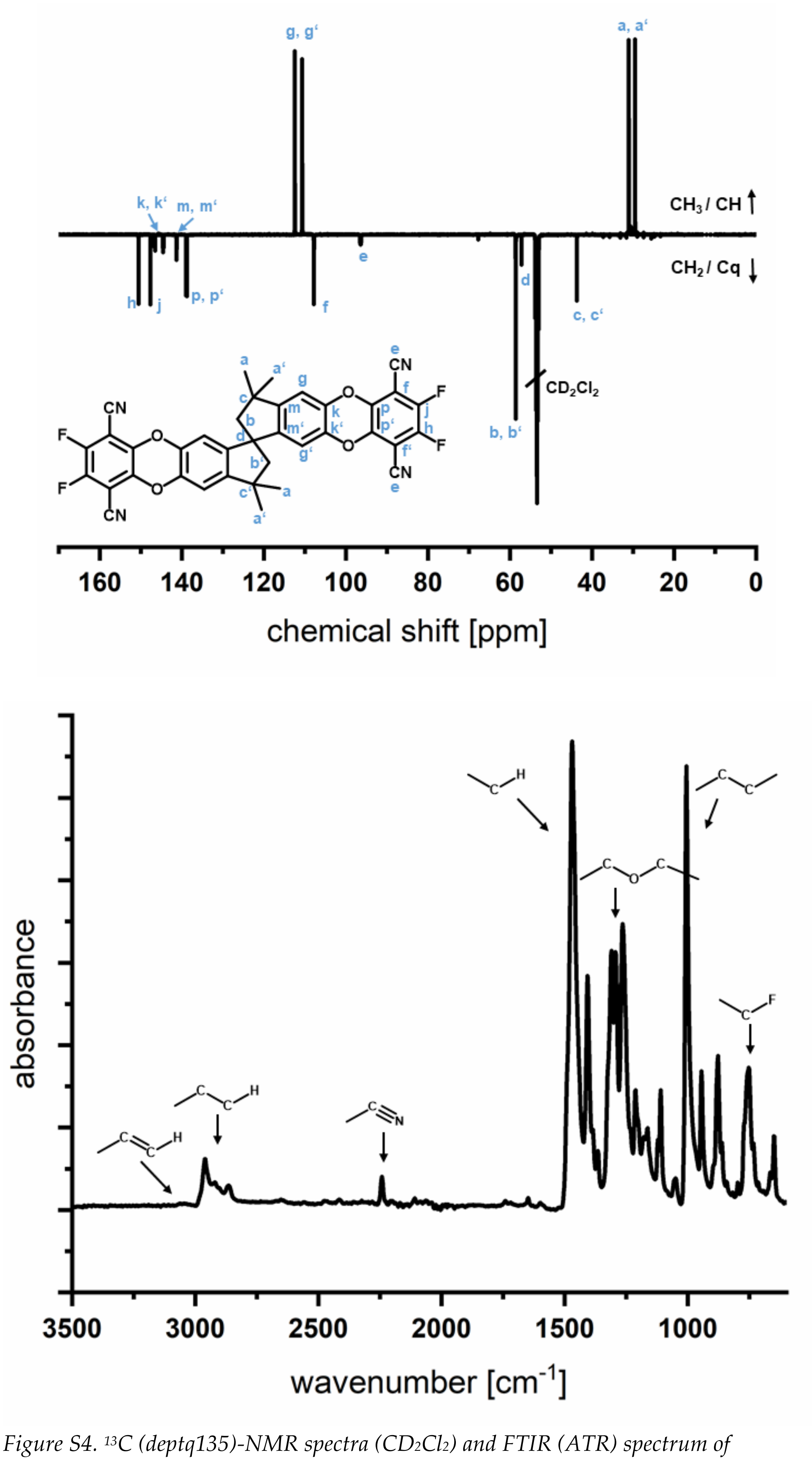

Figure S4. ^{13}C *(deptq135)-NMR spectra (CD_2Cl_2) and FTIR (ATR) spectrum of synthezised trimer for preparation of an alternating copolymer*

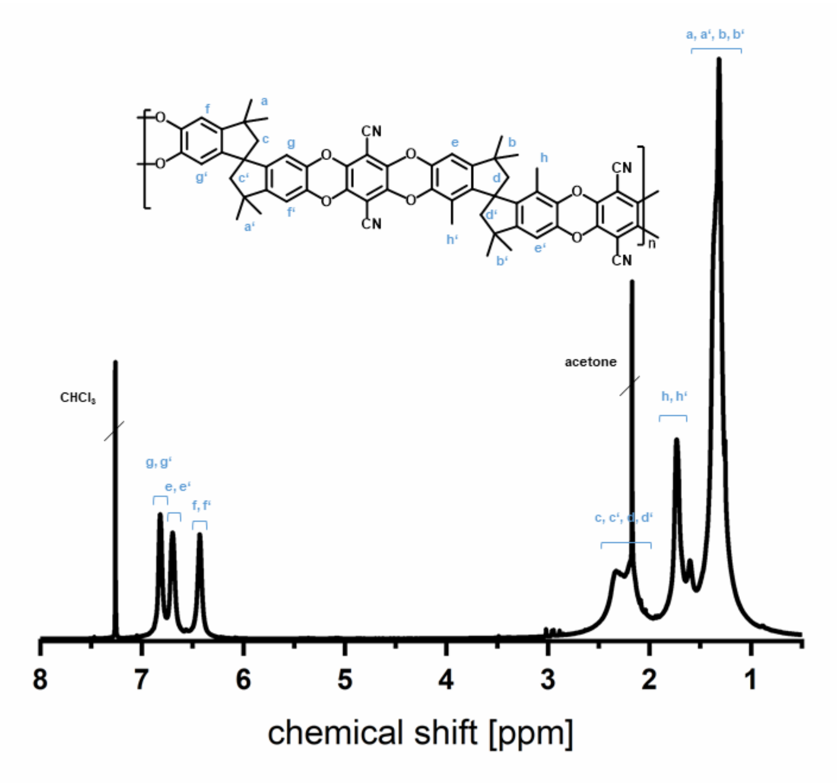

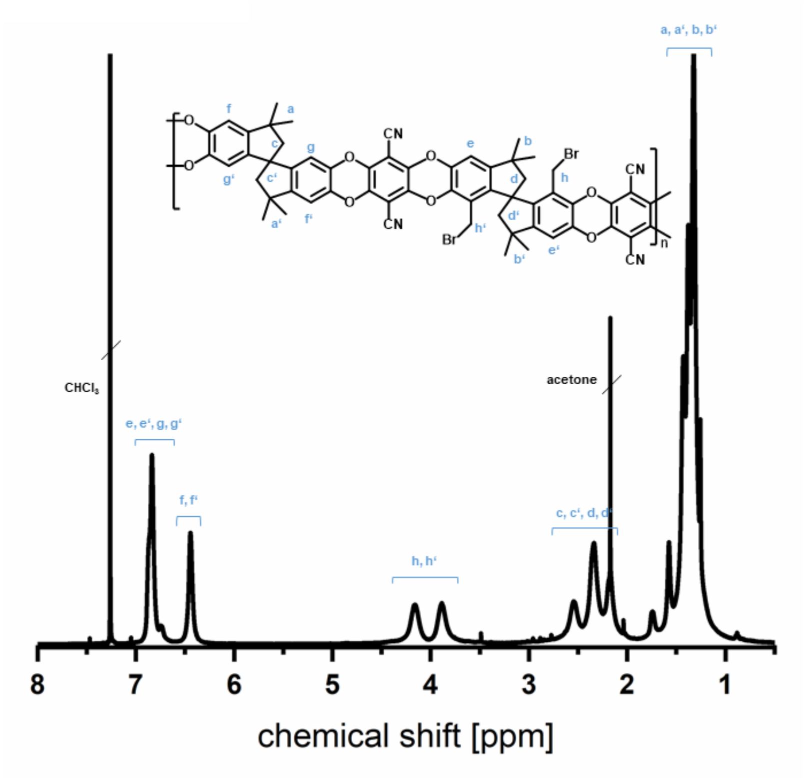

Figure S5. ^{1}H-NMR ($CDCl_3$) of PIM-DMTTSBI-A50 and PIM-Br-DMTTSBI-A50

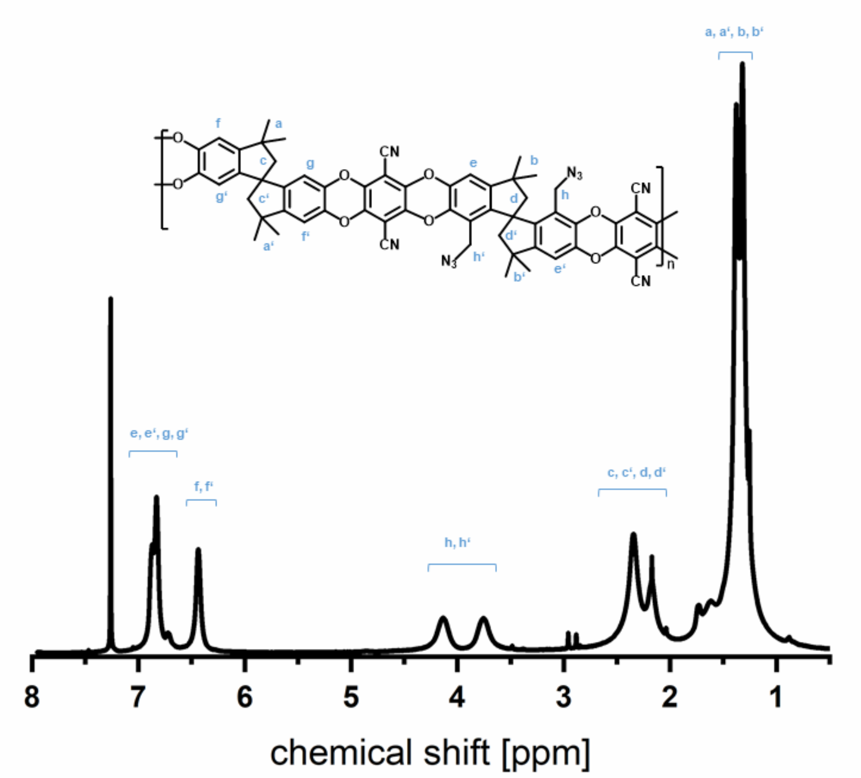

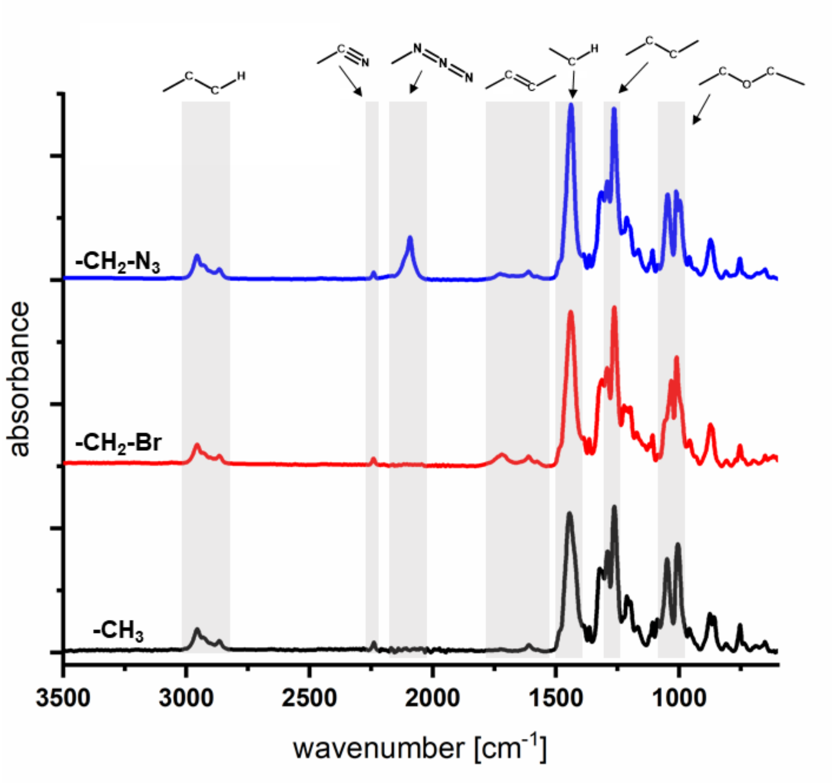

Figure S6. ¹H-NMR (CDCl₃) of AZ-PIM-A50 and FTIR (ATR) spectra of all modification steps of –A50 (black – unmodified, red – brominated, blue – azide modified)

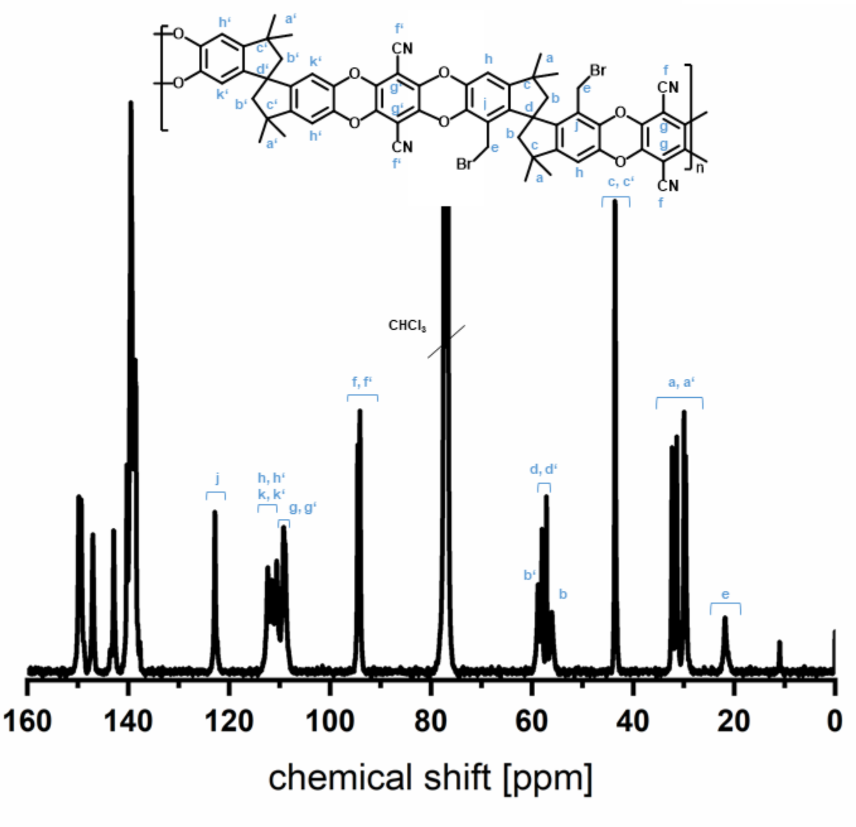

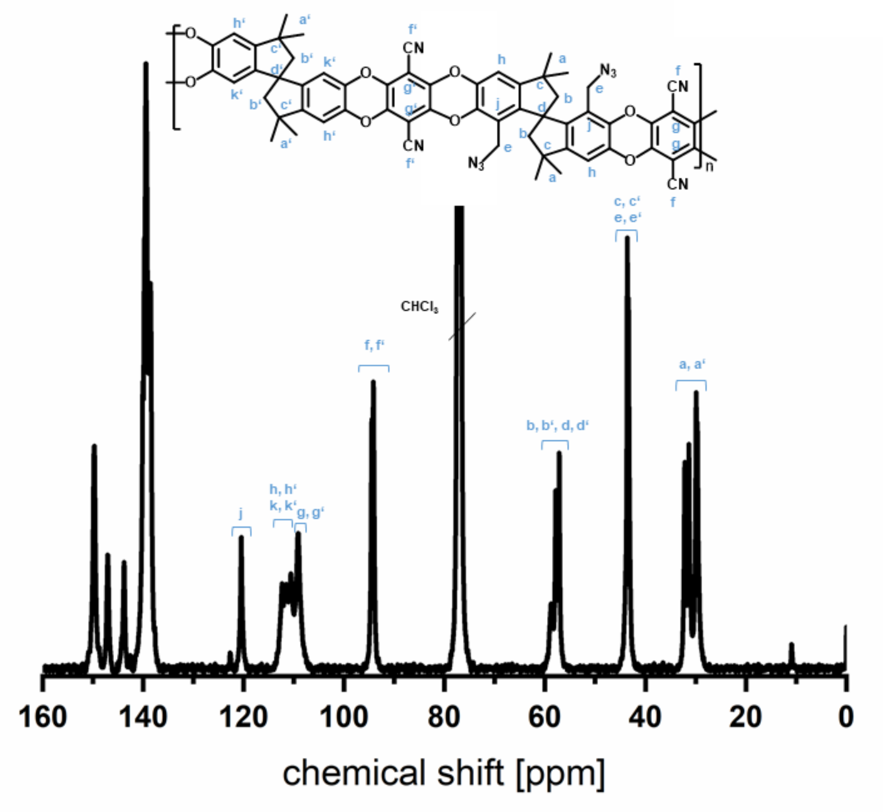

Figure S7. ^{13}C-NMR (CDCl3) IGD-spectra (inverse-gated decoupling) of PIM-Br-DMTTSBI-A50 and AZ-PIM-A50

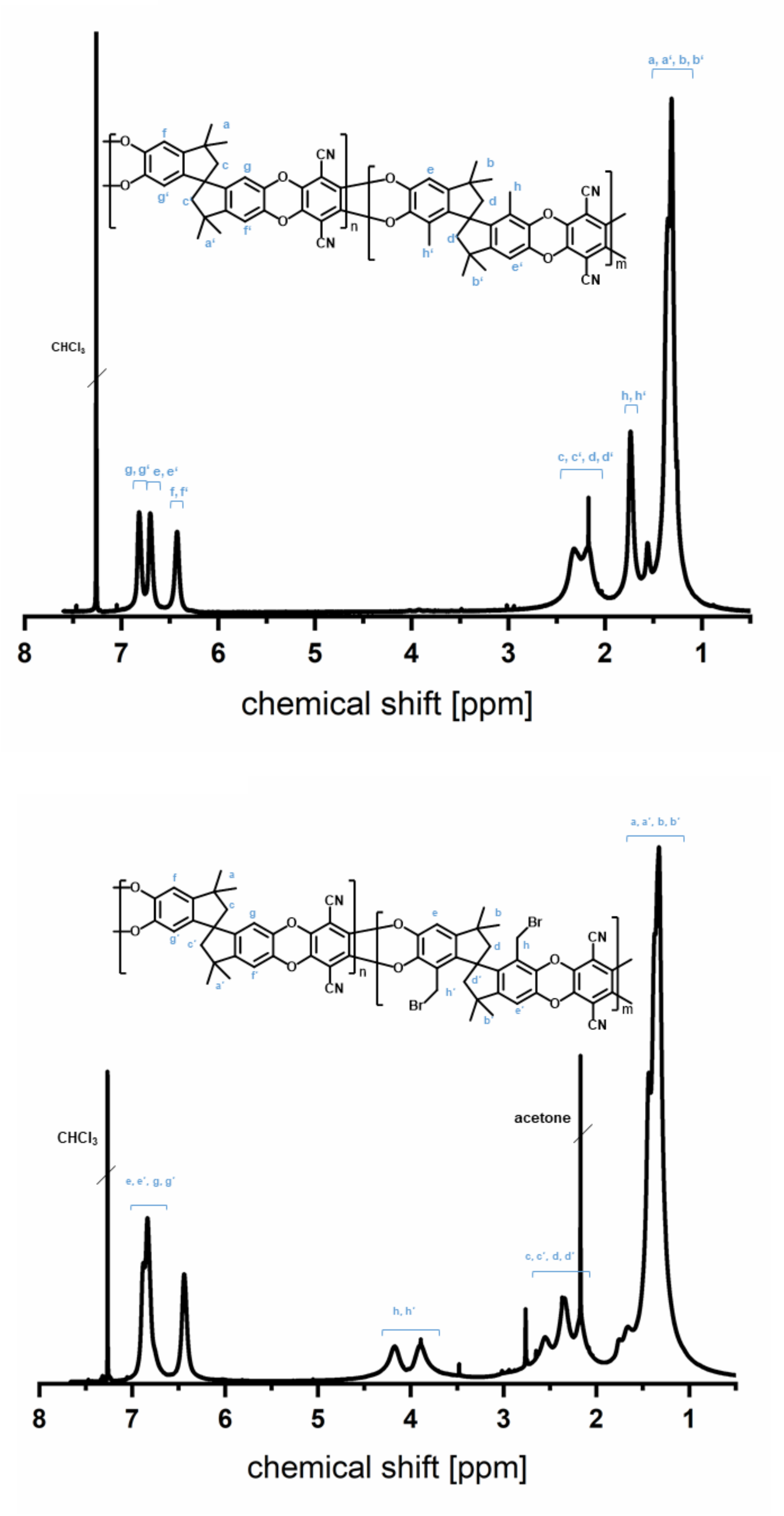

Figure S8. ^{1}H-NMR ($CDCl_3$) of PIM-DMTTSBI-R50 and PIM-Br-DMTTSBI-R50

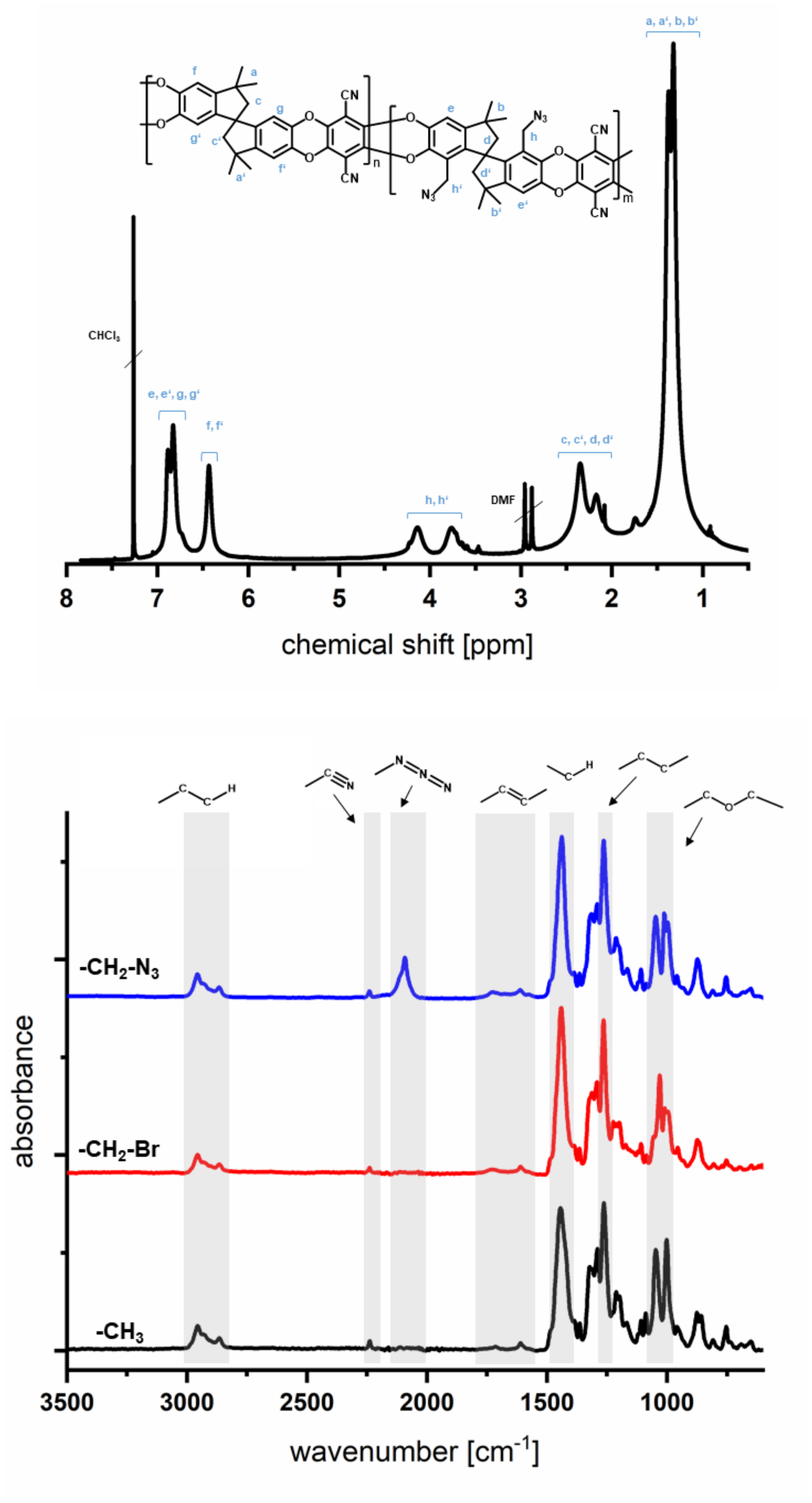

Figure S9. ¹H-NMR (CDCl₃) of AZ-PIM-R50 and FTIR (ATR) spectra of all modification steps of –R50 (black – unmodified, red – brominated, blue – azide modified)

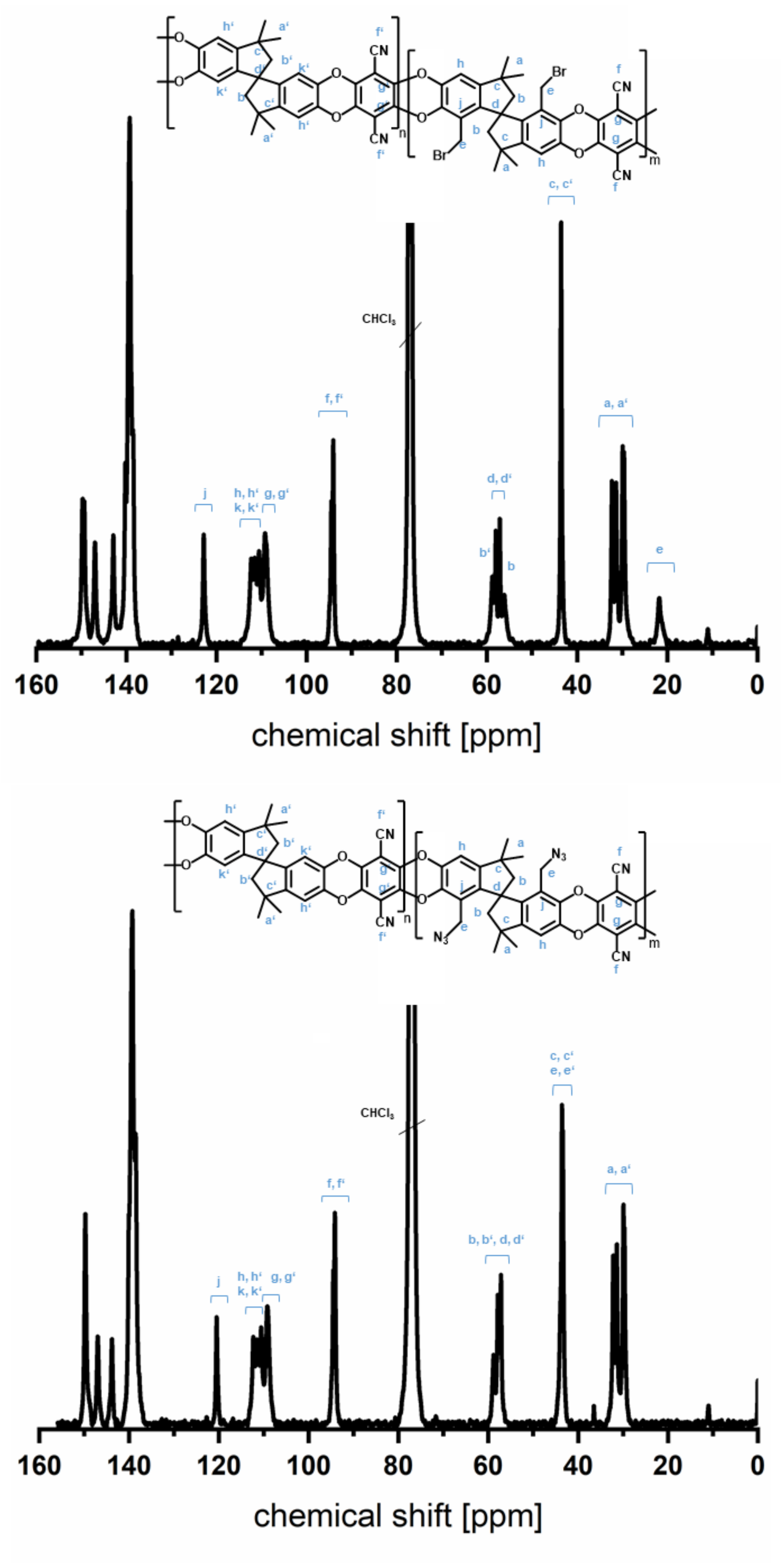

Figure S10. ^{13}C-NMR ($CDCl_3$) IGD-spectra (inverse-gated decoupling) of PIM-Br-DMTTSBI-R50 and AZ-PIM-R50

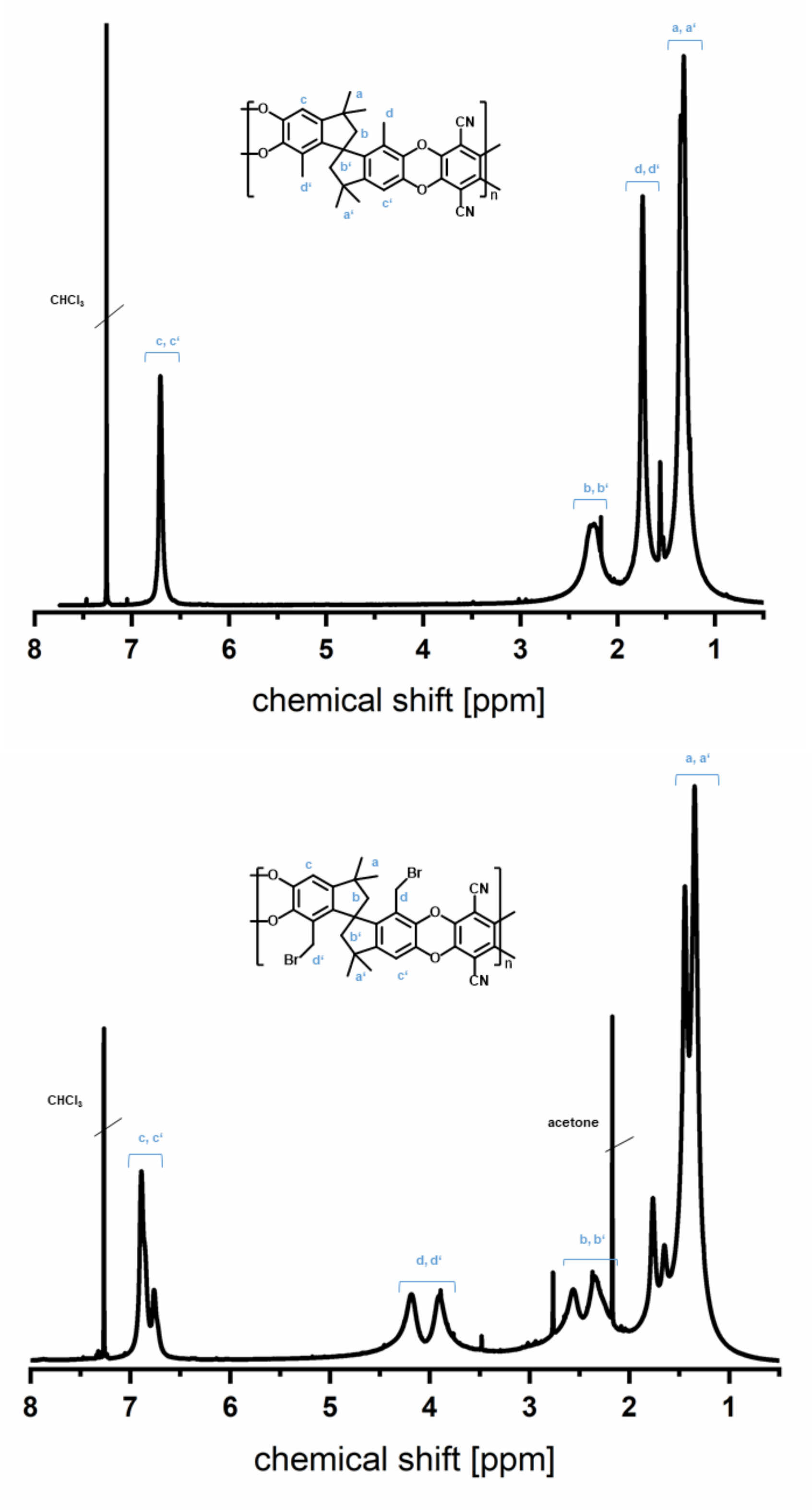

Figure S11. ^{1}H-NMR ($CDCl_3$) of PIM-DMTTSBI-100 and PIM-Br-DMTTSBI-100

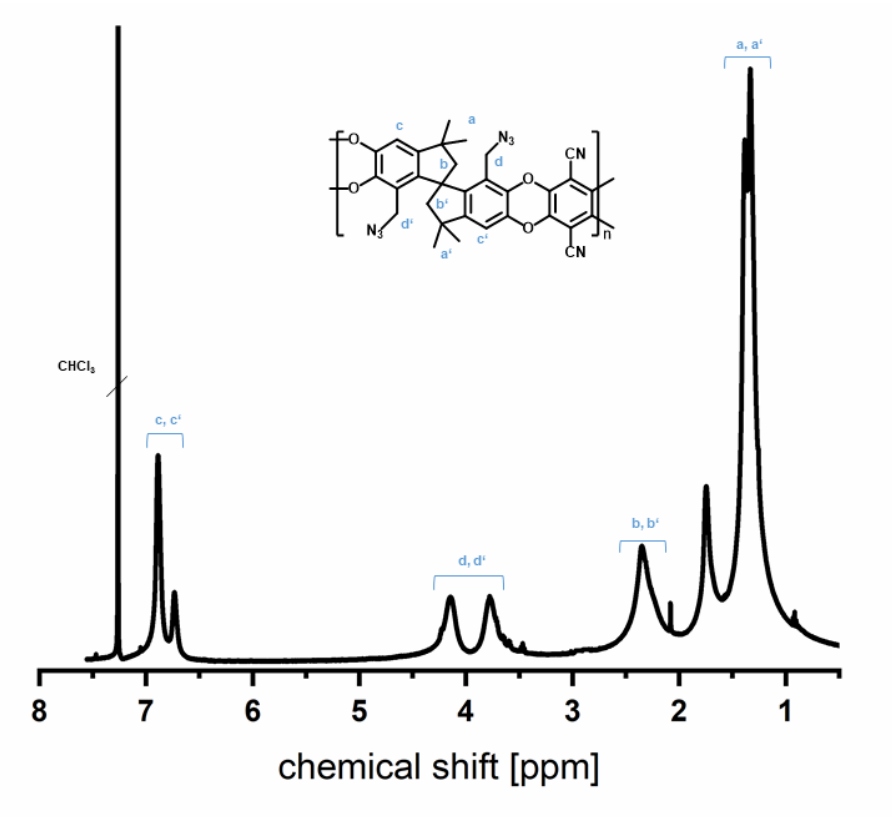

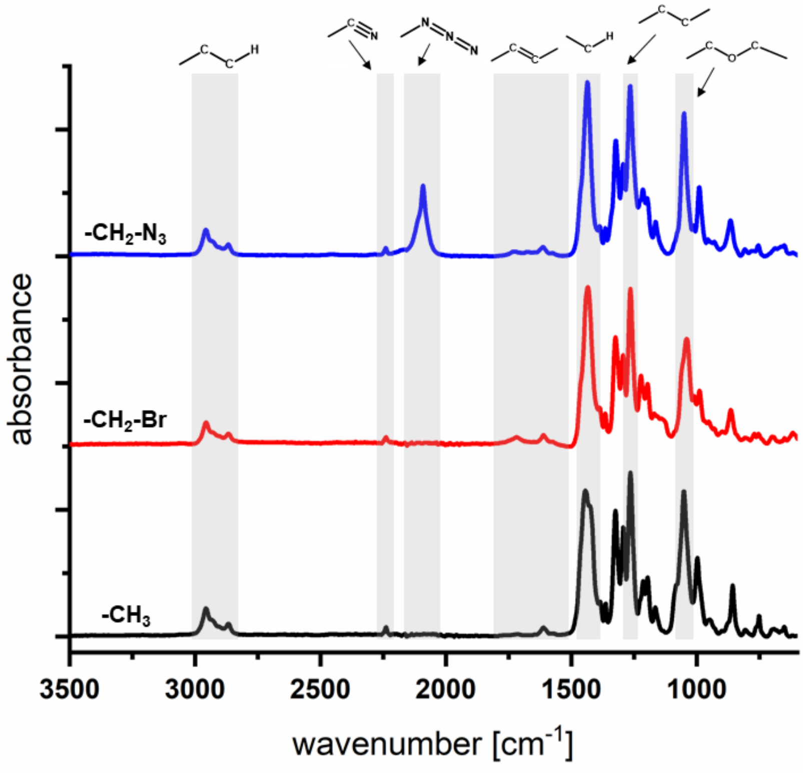

Figure S12. ¹H-NMR (CDCl3) of AZ-PIM-100 and FTIR (ATR) spectra of all modification steps of AZ-PIM–100 (black – unmodified, red – brominated, blue – azide modified)

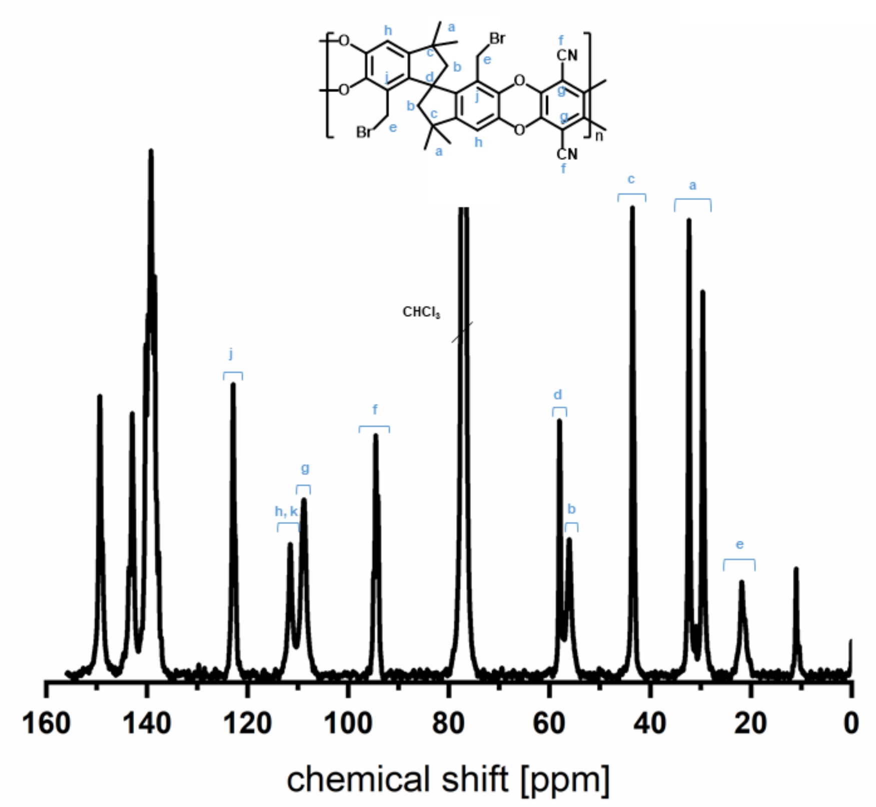

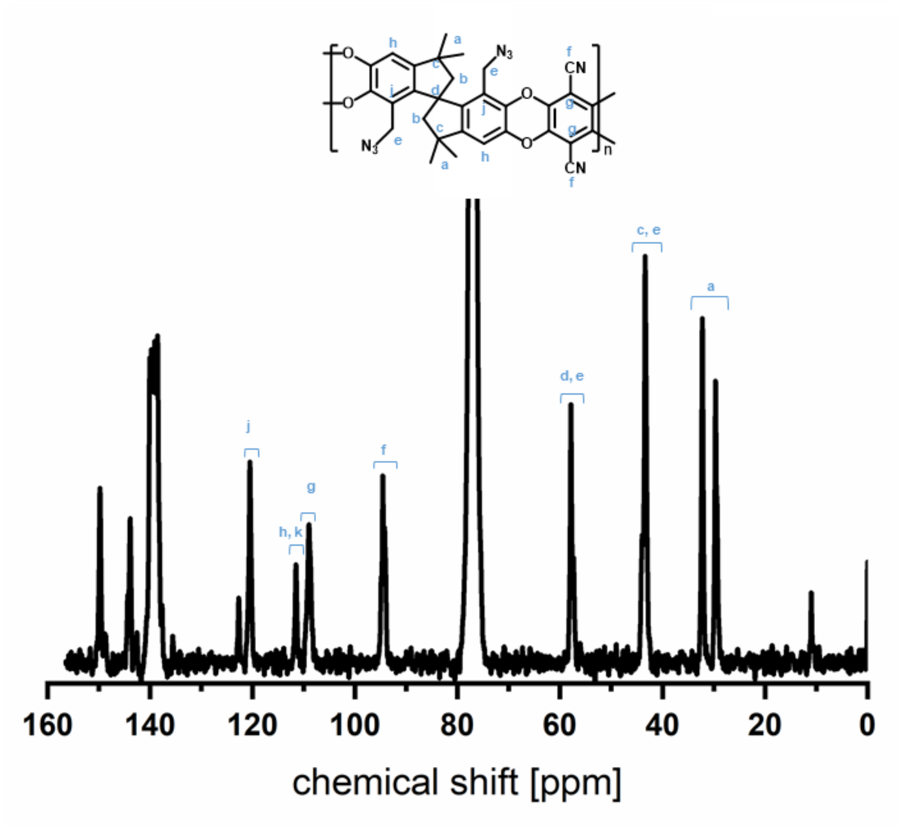

Figure S13. ^{13}C-NMR (CDCl3) IGD-spectra (inverse-gated decoupling) of PIM-Br-DMTTSBI-100 and AZ-PIM-100

**Polymer**	***dn/dc* *[cm³/g]***	***M_w* *(RI)* *[kg/mol]***	***D* *(RI)***	***M_w* *(MALS)* *[kg/mol]***	***D* *(MALS)***	***M_w* *(Visc)* *[kg/mol]***	***D* *(Visc)***
PIM-DMTTSBI-100	*0.190*	157.9	6.4	214.5	4.3	162.8	9.6
PIM-Br-DMTTSBI-100	*0.170*	82.9	3.6	121.7	2.4	95.0	5.9
AZ-PIM-100	*0.107*	28.3	2.4	78.7	1.3	45.8	3.2
PIM-DMTTSBI-R50	*0.154*	204.6	9.7	405.4	2.1	274.3	17.2
PIM-Br-DMTTSBI-R50	*0.171*	86.2	4.3	134.3	2.5	104.5	7.1
AZ-PIM-R50	*0.109*	35.2	3.5	110.5	1.3	50.5	5.8
PIM-DMTTSBI-A50	*0.186*	138.7	5.9	176.7	2.9	176.1	5.6
PIM-Br-DMTTSBI-A50	-	-	-	-	-	-	-
AZ-PIM-A50	*0.115*	63.2	3.3	152.1	1.8	106.6	2.3

Table S1. Results of molecular weight determination by SEC in chloroform

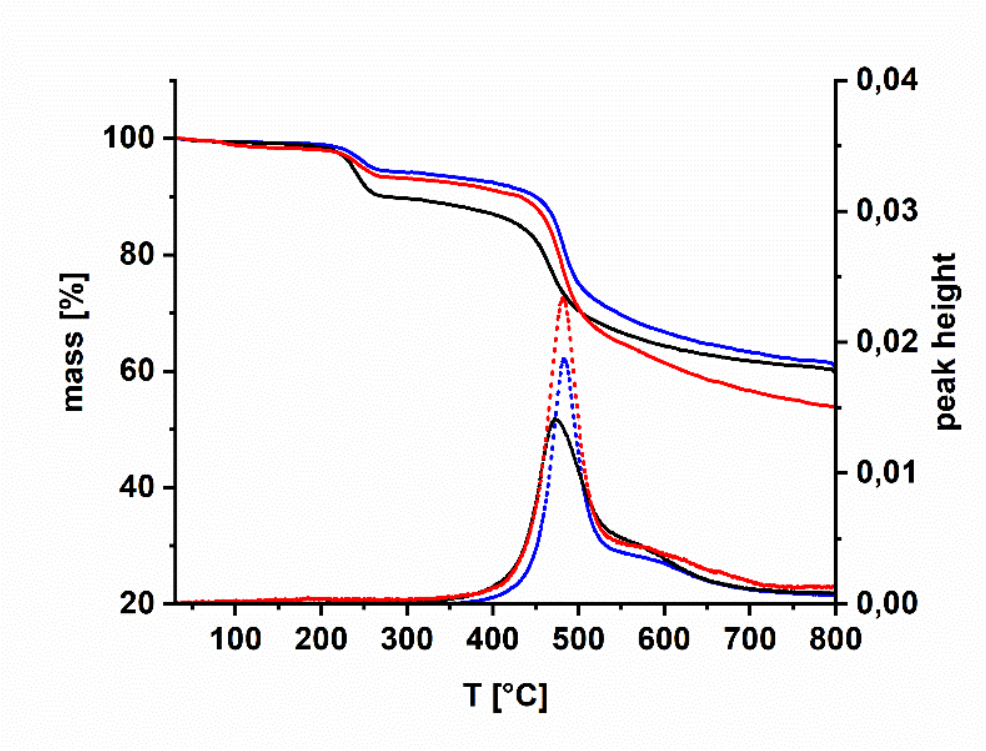

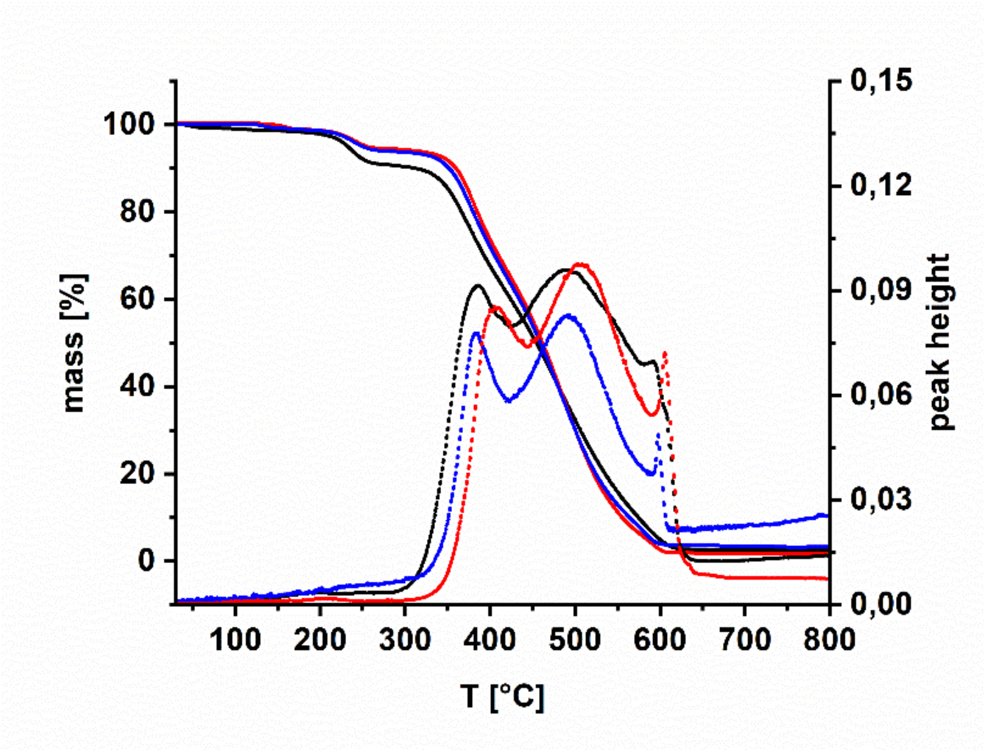

Figure S14. TGA graphs of AZ-PIM-R50 (red), AZ-PIM-A50 (blue) and AZ-PIM-100 (black) in argon (top) and synthetic air (bottom)

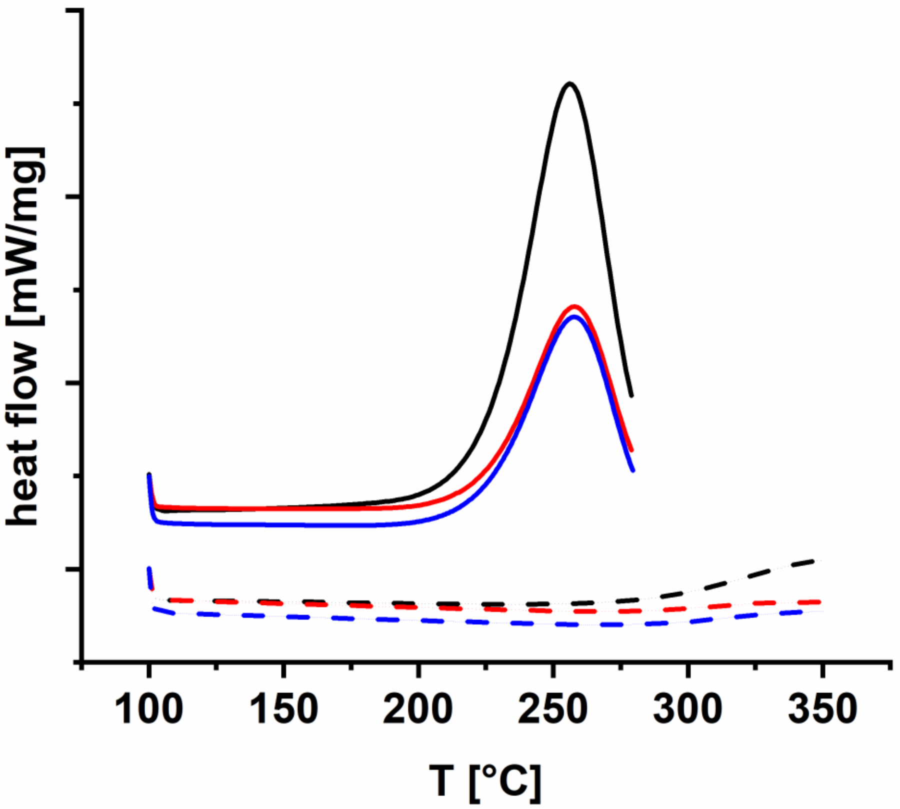

Figure S15. DSC graph of AZ-PIM-R50 (red), AZ-PIM-A50 (blue) and AZ-PIM-100 (black); solid line - first heating, dashed line – second heating

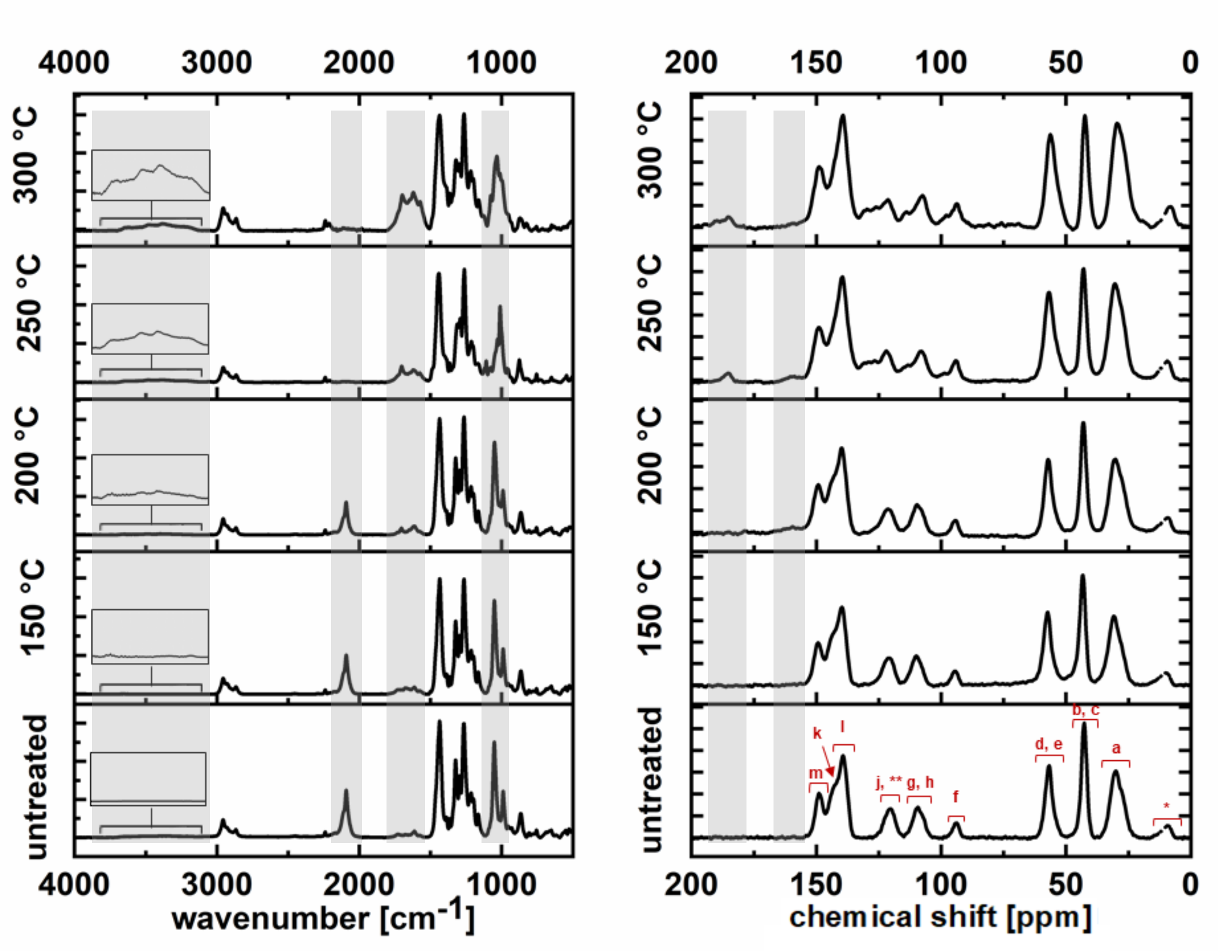

Figure S16. FTIR (left, ATR) and solid-state NMR (right, CP-MAS) spectra of the temperature treated AZ-PIM-100 and suggested structure of cross-linked AZ-PIMs

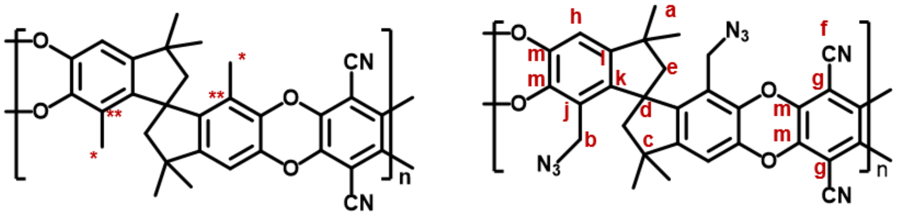

Peaks in FTIR:

3800 – 3100 cm^{-1}:	R-NH-R′	N-H stretching vibrations (primary, secondary amines and imines)
2091 cm^{-1}:	R-N=N=N	asymmetric stretching vibration
1730 – 1550 cm^{-1}:	R-C=O	C=O stretching vibration (1700 – 1730 cm^{-1})
	R,R′-C=N-R′′	C=N stretching vibration (1620 – 1690 cm^{-1})
	R-C=C-R′	stretching vibration (1580 – 1650 cm^{-1})
	R-C-NH-R′	deformation vibration (1580 – 1650 cm^{-1})
1020 – 1080 cm^{-1}:	R-C-O-C-R′	C-O-C stretching vibration

Unassigned peaks in CP-MAS spectra:

158.7 ppm:	R-C=N-R′	imines
184.8 ppm:	R-C=O-R′	quinone (carbonyl)

			permeability (Barrer)					$\alpha_{P(x)/P(y)}$			
**Polymer**	*T* [°C]	*ρ* [g/cm³]	N_2	CO_2	CH_4	H_2	O_2	CO_2/N_2	CO_2/CH_4	H_2/CH_4	O_2/N_2
**AZ-PIM-100**	30	1.143	147	2894	245	1206	422	19.8	11.8	4.9	2.9
	150	1.168	67	1483	116	776	233	22.0	12.8	6.7	3.5
	200	1.122	200	4239	308	1859	614	21.2	13.7	6.0	3.1
	250	1.108	221	4551	298	2156	694	20.6	15.3	7.2	3.1
	300	1.126	241	4977	351	2218	738	20.6	14.2	6.3	3.1
**AZ-PIM-R50**	30	1.116	237	4246	384	1863	658	17.9	11.1	4.9	2.8
	150	1.145	83	1846	126	1293	293	22.2	14.6	10.2	3.4
	200	1.112	242	4709	368	3009	814	19.5	12.8	8.2	3.4
	250	1.078	312	5860	476	2475	876	18.8	12.3	5.2	2.8
	300	1.118	301	6042	479	2489	885	20.1	12.6	5.2	2.9
**AZ-PIM-A50**	30	1.099	192	3879	331	1507	558	20.2	11.7	4.6	2.9
	150	1.101	166	3422	264	1517	518	20.6	12.9	5.7	3.1
	200	1.078	397	7206	704	2663	1051	18.2	10.2	3.8	2.7
	250	1.078	473	8712	839	3095	1254	18.4	10.4	3.7	2.6
	300	1.079	527	8868	911	3379	1341	16.8	9.7	3.7	2.5

Table S2. Permeability of different gases of the freshly prepared untreated AZ-PIMs and after treatment at different temperature

		solubility (cm³/cm³ cmHg)					$\alpha_{S(x)/S(y)}$			
**Polymer**	*T* [°C]	N_2	CO_2	CH_4	H_2	O_2	CO_2/N_2	CO_2/CH_4	H_2/CH_4	O_2/N_2
**AZ-PIM-100**	30	0.032	0.575	0.125	0.006	0.034	18.14	4.60	0.050	1.09
	150	0.028	0.558	0.121	0.006	0.034	19.71	4.61	0.046	1.19
	200	0.039	0.714	0.164	0.007	0.040	18.49	4.34	0.045	1.03
	250	0.042	0.779	0.175	0.008	0.045	18.64	4.46	0.043	1.06
	300	0.042	0.778	0.177	0.007	0.045	18.39	4.39	0.042	1.07
**AZ-PIM-R50**	30	0.042	0.708	0.169	0.009	0.043	16.89	4.18	0.052	1.03
	150	0.028	0.606	0.114	0.007	0.036	21.35	5.32	0.064	1.27
	200	0.040	0.757	0.165	0.011	0.044	18.95	4.58	0.066	1.10
	250	0.045	0.829	0.195	0.010	0.052	18.26	4.26	0.053	1.14
	300	0.045	0.798	0.186	0.010	0.047	17.76	4.30	0.052	1.06

		solubility (cm^3/cm^3 cmHg)					α S(x)/S(y)			
**Polymer**	*T* *[°C]*	*N_2*	*CO_2*	*CH_4*	*H_2*	*O_2*	*CO_2/N_2*	*CO_2/CH_4*	*H_2/CH_4*	*O_2/N_2*
**AZ-PIM-A50**	30	0.035	0.643	0.146	0.007	0.039	18.37	4.42	0.047	1.11
	150	0.038	0.650	0.150	0.007	0.040	17.32	4.33	0.049	1.06
	200	0.038	0.673	0.161	0.009	0.042	17.52	4.17	0.054	1.09
	250	0.038	0.703	0.167	0.008	0.041	18.32	4.22	0.046	1.08
	300	0.044	0.723	0.185	0.009	0.046	16.51	3.90	0.049	1.06

Table S3. Solubility coefficients of different gases of freshly prepared untreated AZ-PIMs and after treatment at different temperatures

		diffusivity ($x10^{-6}$ cm^2/s)					α D(x)/D(y)			
**Polymer**	*T* *[°C]*	*N_2*	*CO_2*	*CH_4*	*H_2*	*O_2*	*CO_2/N_2*	*CO_2/CH_4*	*H_2/CH_4*	*O_2/N_2*
**AZ-PIM_100**	30	0.462	0.504	0.196	19.732	1.225	1.09	2.57	100.57	2.65
	150	0.238	0.266	0.096	13.816	0.690	1.12	2.78	144.22	2.90
	200	0.519	0.595	0.188	25.040	1.541	1.15	3.17	133.54	2.97
	250	0.531	0.585	0.170	28.723	1.559	1.10	3.43	168.56	2.94
	300	0.571	0.640	0.198	30.121	1.633	1.12	3.23	151.97	2.86
**AZ-PIM-R50**	30	0.567	0.601	0.227	21.219	1.522	1.06	2.65	93.43	2.69
	150	0.294	0.305	0.111	18.174	0.815	1.04	2.74	163.58	2.78
	200	0.605	0.623	0.223	27.589	1.850	1.03	2.79	123.61	3.06
	250	0.687	0.707	0.245	25.255	1.704	1.03	2.89	103.25	2.48
	300	0.670	0.758	0.258	26.364	1.870	1.13	2.94	102.23	2.79
**AZ-PIM-A50**	30	0.550	0.604	0.227	22.225	1.442	1.10	2.66	97.85	2.62
	150	0.443	0.527	0.176	20.817	1.300	1.02	2.99	118.07	2.94
	200	1.033	1.072	0.436	31.112	2.523	1.04	2.46	71.29	2.44
	250	1.234	1.240	0.502	40.029	3.028	1.00	2.47	79.74	2.45
	300	1.203	1.227	0.491	37.427	2.894	1.02	2.50	76.17	2.40

Table S4. Diffusivity coefficients of different gases of the freshly prepared untreated AZ-PIMs and after treatment at different temperature

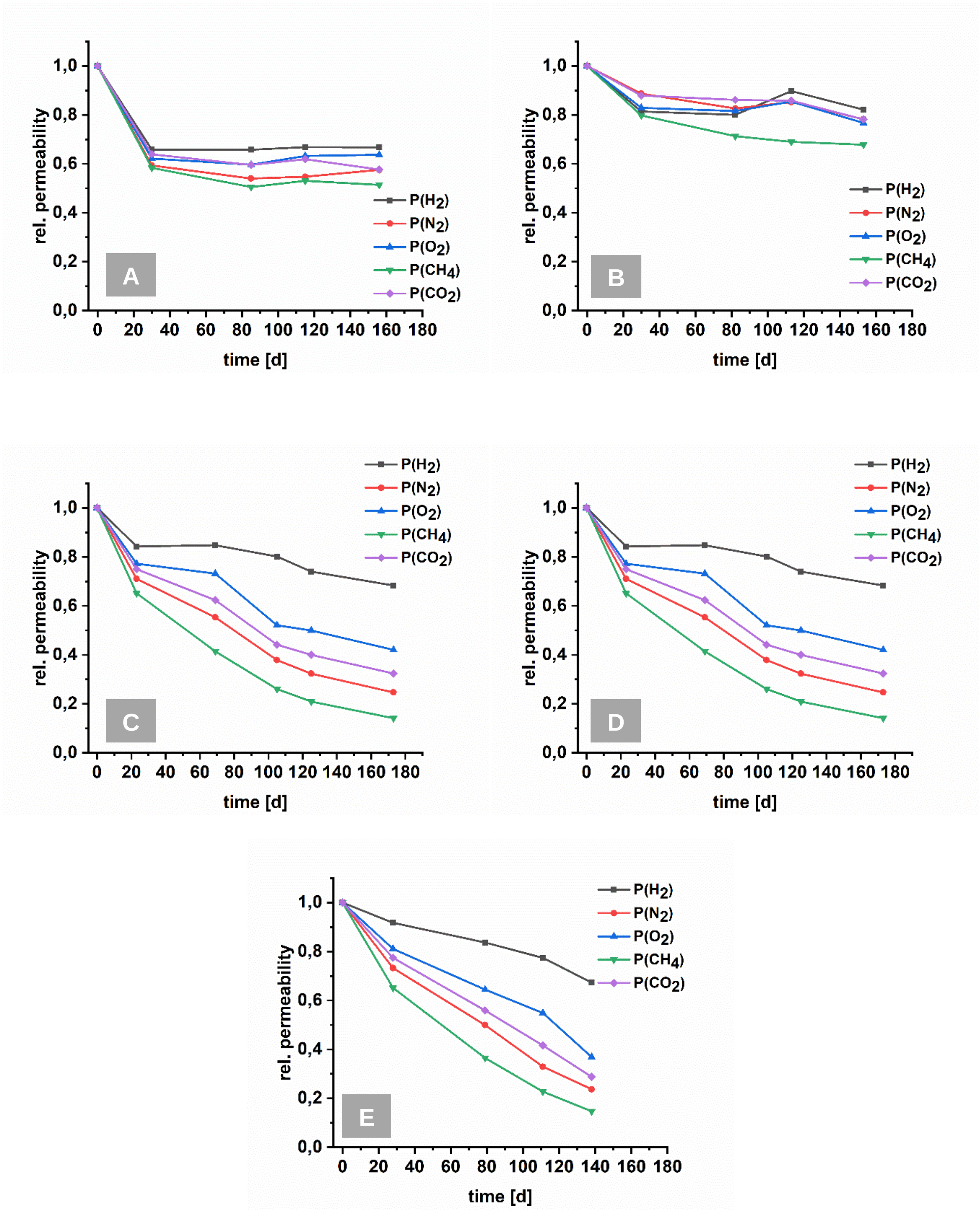

Figure S17. Relative permeability depending on time of AZ-PIM-100, treated at different temperatures (A: 30 °C – no treatment, B: 150 °C, C: 200 °C, D: 250 °C, E: 300 °C)

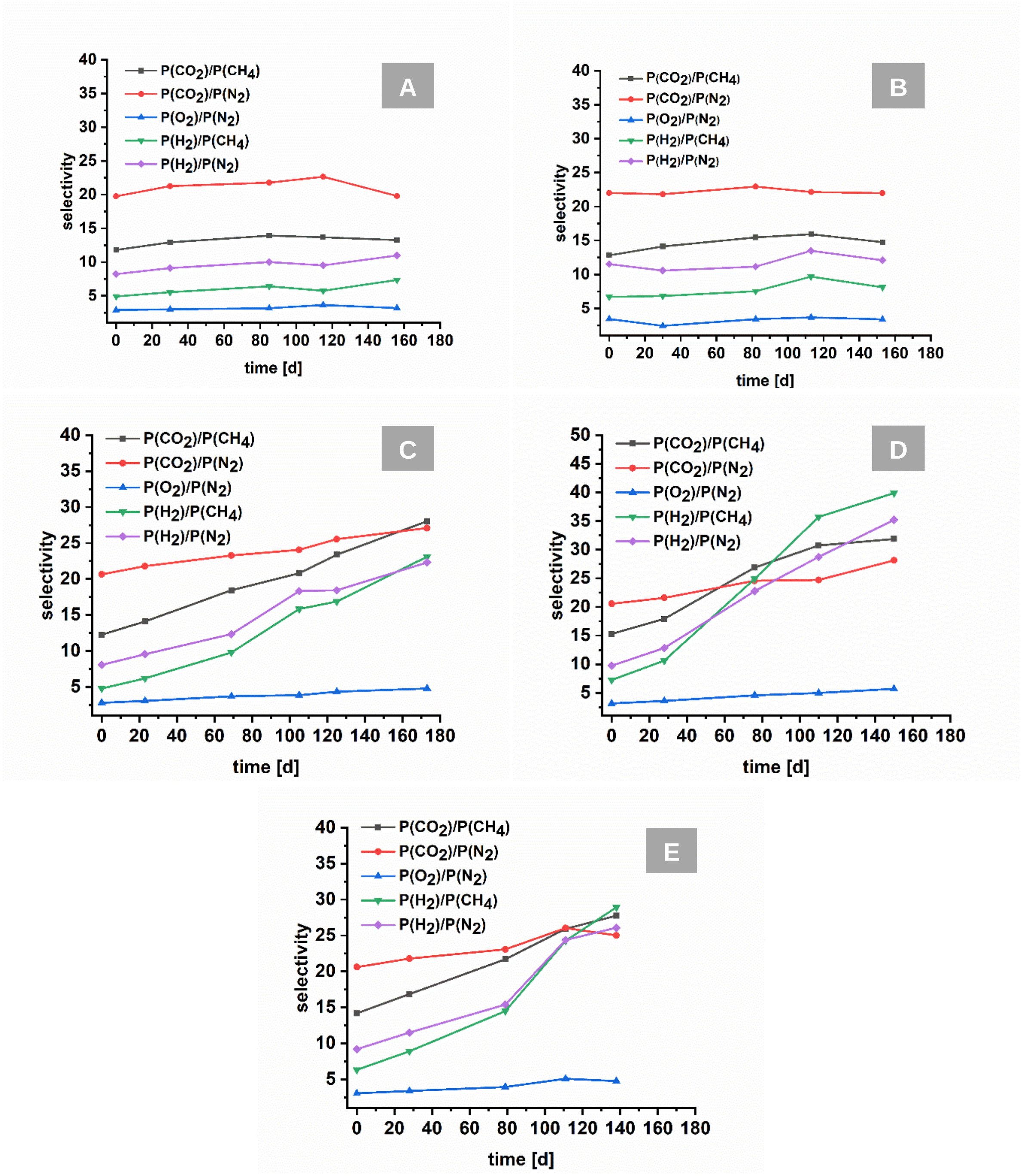

Figure S18. Development of selectivity of different gas pairs depending on time measured with AZ-PIM-100, treated at different temperatures (A: 30 °C – no treatment, B: 150 °C,

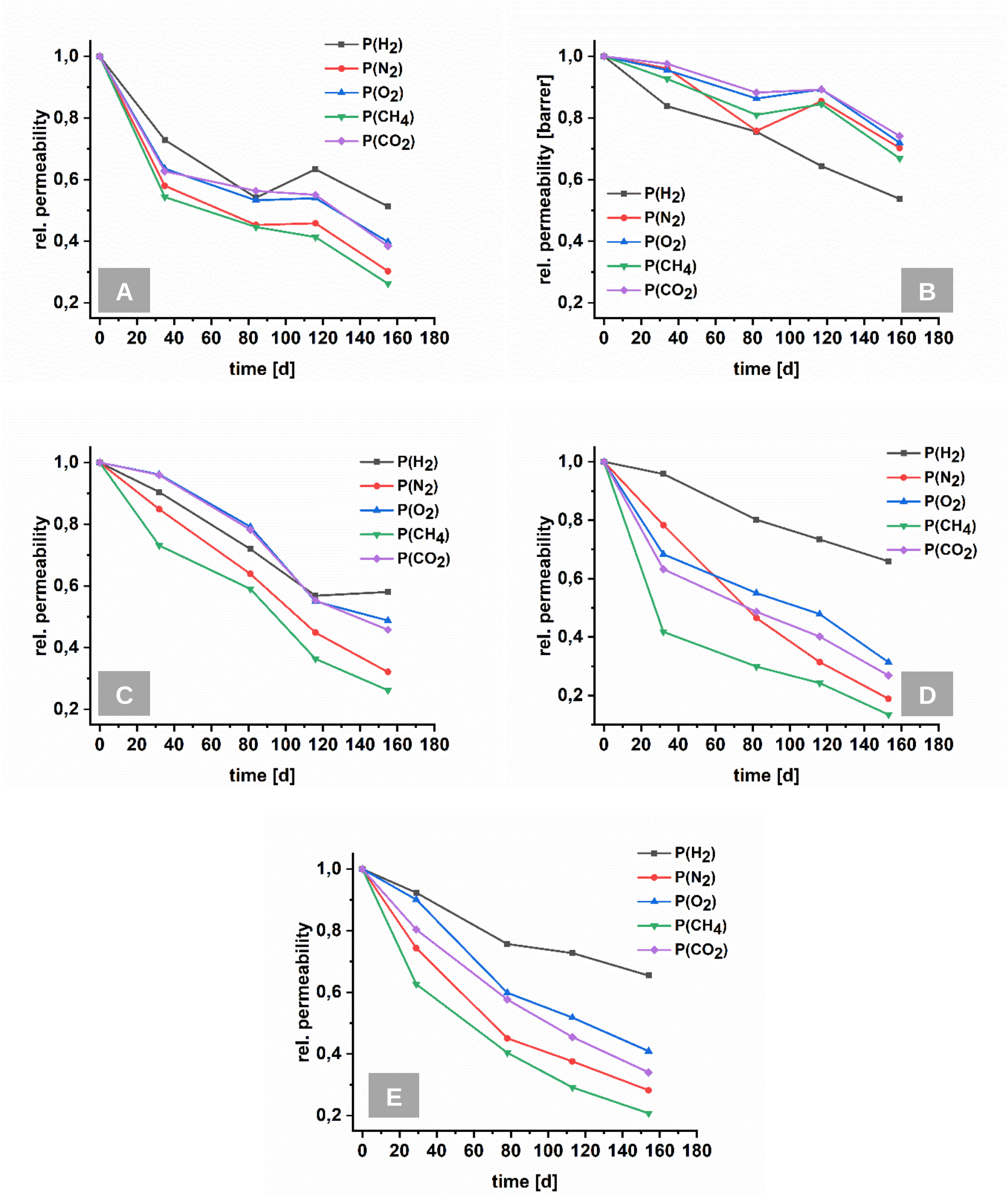

Figure S19. Relative permeability depending on time of AZ-PIM-R50, treated at different temperatures (A: 30 °C – no treatment, B: 150 °C, C: 200 °C, D: 250 °C, E: 300 °C)

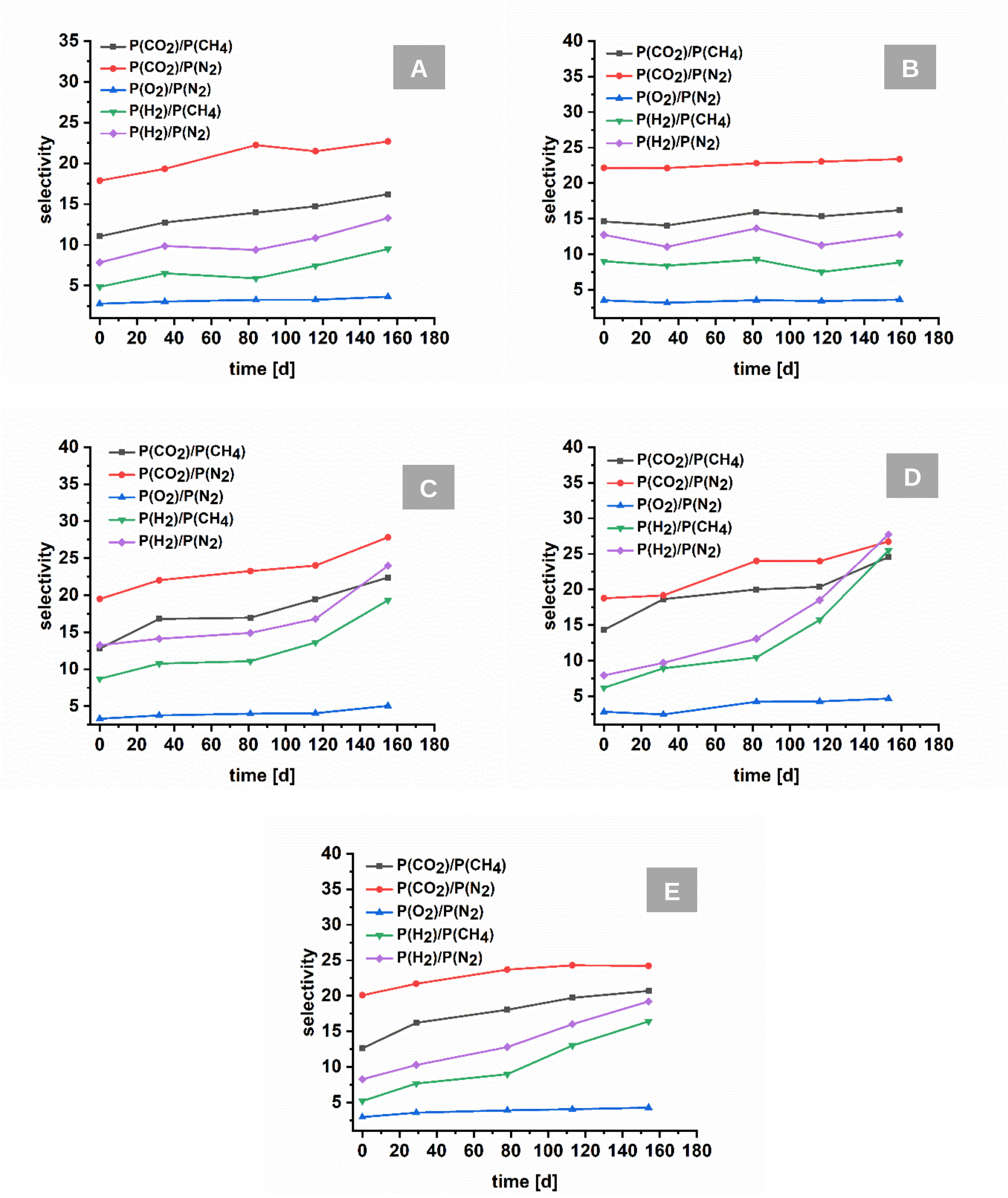

Figure S20. Development of selectivity of different gas pairs depending on time of AZ-PIM-R50, treated at different temperatures (A: 30 °C – no treatment, B: 150 °C, C: 200 °C, D: 250 °C, E: 300 °C)

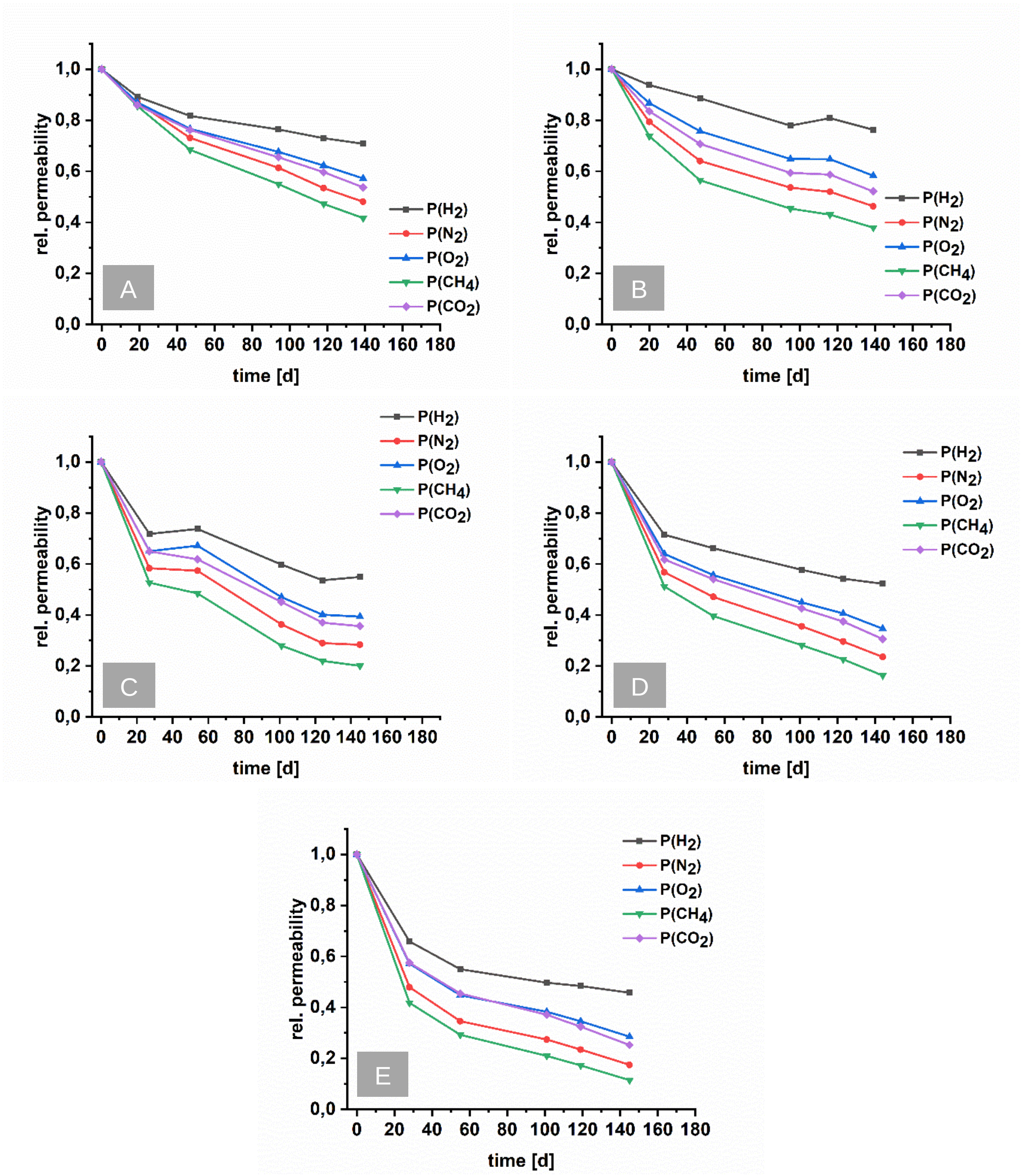

Figure S21. Relative permeability depending on time of AZ-PIM-A50, treated at different temperatures (A: 30 °C – no treatment, B: 150 °C, C: 200 °C, D: 250 °C, E: 300 °C)

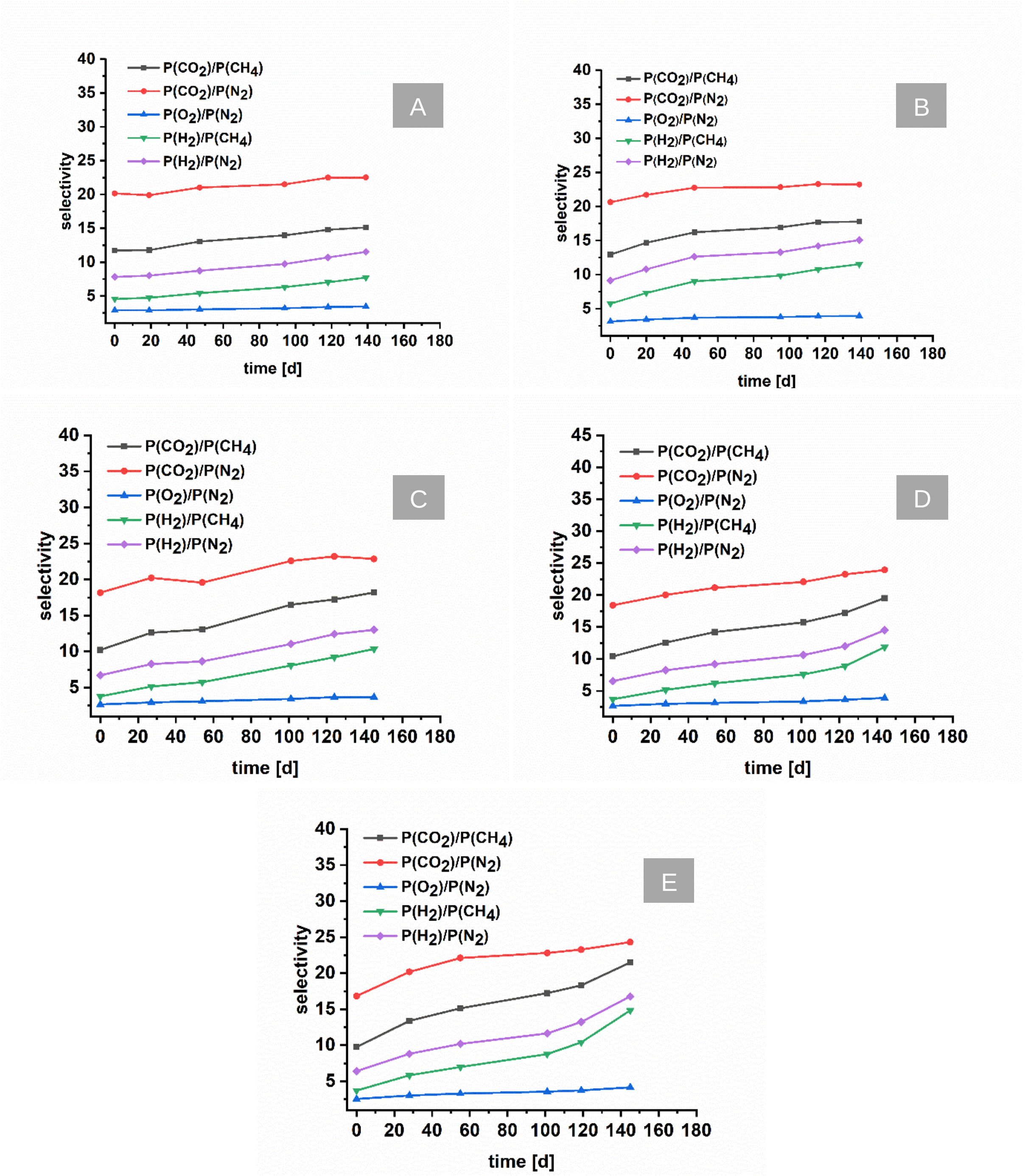

Figure S22. Development of selectivity of different gas pairs depending on time of AZ-PIM-A50, treated at different temperatures (A: 30 °C – no treatment, B: 150 °C, C: 200 °C, D: 250 °C, E: 300 °C)

Additionally, the position of the materials before and after aging is presented in the graph, illustrated with the help of a line. Due to physical aging permeability decreases. Therefore, the points with lower permeability correspond to the aged materials after approximately 5 month.

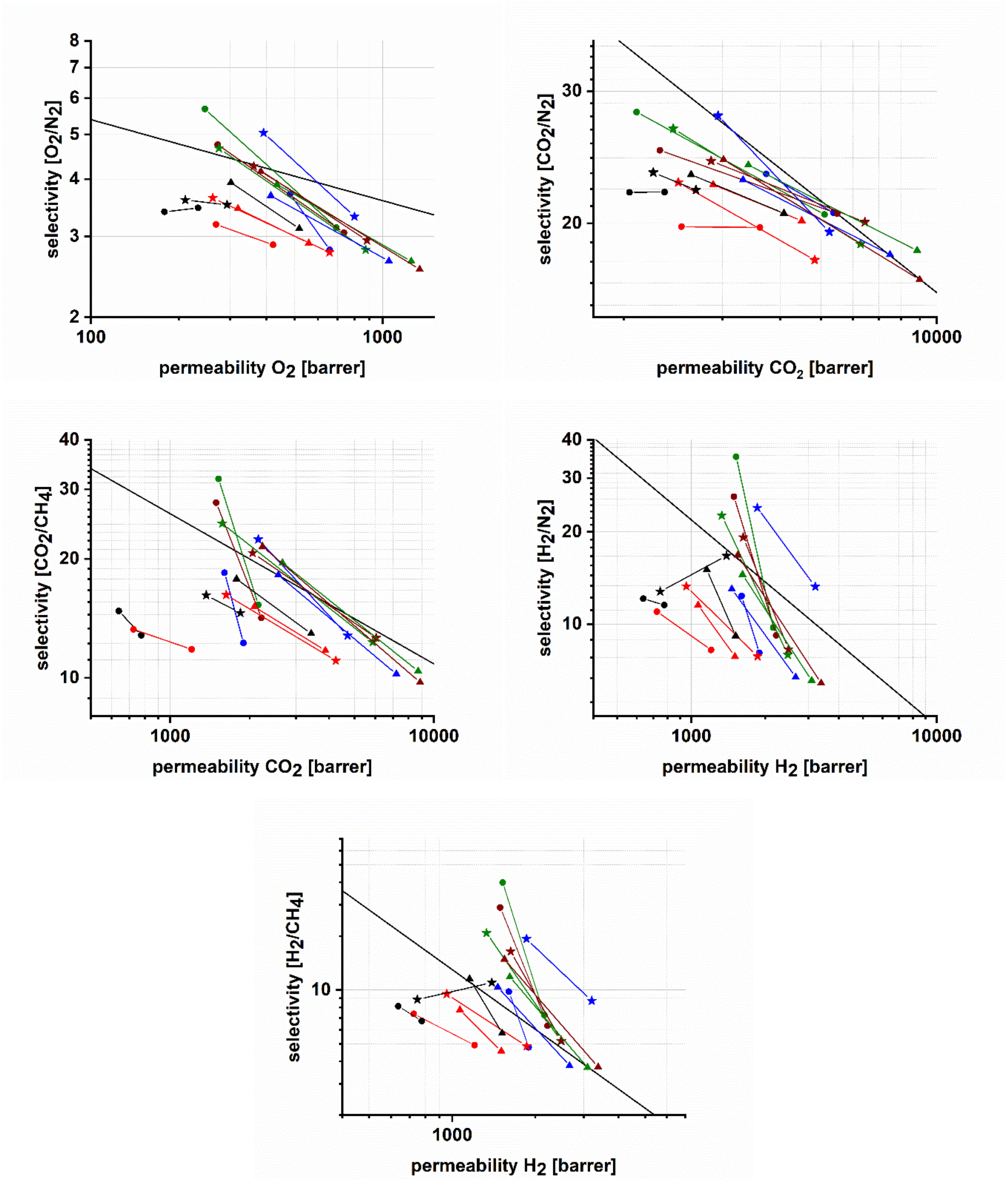

Figure S23. Robeson-Plots of different gas pairs of the three temperature treated Azide-PIMs (circles – AZ-PIM-100, stars – AZ-PIM-R50, triangle – AZ-PIM-A50; red – not temperature treated, black – 150 °C, blue – 200 °C, green – 250 °C, 300 °C – brown)

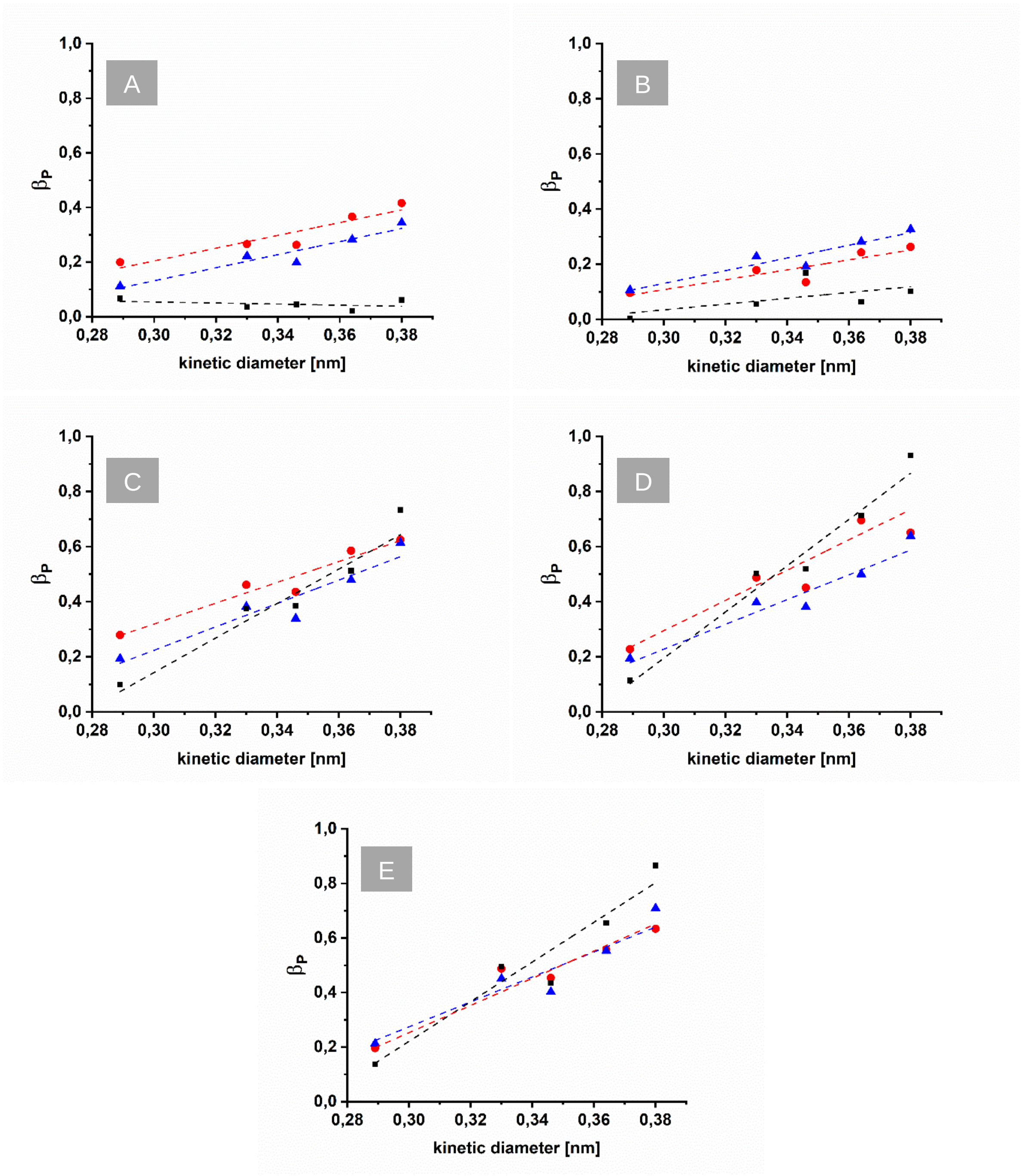

Figure S24. Change of aging rates (βp) depending on the kinetic diameter of tested gases and for different temperature treatments (red – AZ-PIM-R50, blue – AZ-PIM-A50, black – AZ-PIM-100; A: not temperature treated, B: 150C, C: 200°C, D: 250 °C, E: 300°C)
